# Miniaturized Acoustic Sensing Platform for Spatial Mapping of Ultrasonic Fields in Small-Diameter Tube Bundles

**DOI:** 10.3390/s26144505

**Published:** 2026-07-15

**Authors:** Luiz Artur dos Santos da Silva, André Jackson Ramos Simões, Vitor Leão Filardi, Vitor Pinheiro Ferreira, Geydison Gonzaga Demetino, Luiz Carlos Simões Soares Júnior, Leandro do Rozário Teixeira, Lucas Gomes Pereira, Leonardo Rafael Teixeira Cotrim Gomes, Germano Pinto Guedes, Marcus Vinícius Santos da Silva, Juliane Grasiela de Carvalho Gomes, Pedro Eduardo Gonçalves Oliveira, Luís Gustavo Macêdo West, Fábio Oliveira de Mattos, André Luiz Andrade Simões, Iuri Muniz Pepe

**Affiliations:** 1 Center of Exact and Technological Sciences, Federal University of Recôncavo of Bahia, Cruz das Almas 44380-000, BA, Brazil; luiz.atr@ufrb.edu.br (L.A.d.S.d.S.); simoeslc@gmail.com (L.C.S.S.J.); cotrim@ufrb.edu.br (L.R.T.C.G.); 2 Physics Institute, Federal University of Bahia, Barão de Jeremoabo Road, s/n, Salvador 40170-115, BA, Brazil; andrejacksonrs@gmail.com (A.J.R.S.); vitorpferreira@gmail.com (V.P.F.); jdemetino@gmail.com (G.G.D.); leandro.r.teixeira@gmail.com (L.d.R.T.); lucas.gomes.lapo@gmail.com (L.G.P.); germano@uefs.br (G.P.G.); marcus.sansil@gmail.com (M.V.S.d.S.); juliane.gomes@ufba.br (J.G.d.C.G.); pedrooliveirafq@gmail.com (P.E.G.O.); lapo.if@gmail.com (I.M.P.); 3 Electronics Department, Federal Institute of Bahia, Salvador 40301-015, BA, Brazil; mrfilardi@gmail.com; 4 Environmental Engineering Department, Federal University of Bahia, Prof. Aristides Novis Road, n° 02, Federação, Salvador 40210-630, BA, Brazil; luis.west@ufba.br; 5 CENPES-Research, Development and Innovation Institute, Petrobras, Rio de Janeiro 21941-598, RJ, Brazil; fabiomattos@petrobras.com.br

**Keywords:** ultrasonic cavitation, acoustic measurement, miniaturized acoustic probe, confined acoustic fields, shell-and-tube heat exchangers, spatial mapping

## Abstract

**Highlights:**

**What are the main findings?**

- Development of a resilient miniaturized sensor: A waterproof, piston-based acoustic probe with a 7 mm external diameter and a 3.5 mm epoxy-encapsulated piezoelectric element was successfully developed to operate inside narrow 8 mm tubes under harsh ultrasonic cavitation fields.
- Spatial ultrasound mapping: The developed probe enabled spatial mapping of the relative acoustic response amplitude and dominant frequency inside a U-tube heat-exchanger bundle.

**What are the implications of the main findings?**

- Optimized experimental efficiency: The proposed reduced 24-point sampling grid decreases experimental effort while preserving the key spatial trends needed for a preliminary comparison of transducer configurations.
- Enhanced ultrasonic cleaning configuration: The results provide guidance for ultrasonic transducer arrangement and acoustic field diagnosis in confined tube-bundle geometries, aimed at fouling prevention and cleaning in heat exchangers.

**Abstract:**

Shell-and-tube heat exchangers often operate under harsh conditions that induce fouling, leading to loss of thermal efficiency, production downtime, and increased maintenance costs. Conventional cleaning procedures generally require scheduled or unscheduled shutdowns, with direct operational and financial impacts. In this context, ultrasonic cavitation has been investigated as a strategy for fouling prevention and equipment cleaning, with the potential to reduce cleaning downtime or support in-service mitigation strategies. This work presents the development of an acquisition platform based on a miniaturized, waterproof acoustic probe designed for operation inside 8 mm tubes under cavitating ultrasonic fields, with the goal of experimentally mapping the relative acoustic response amplitude and dominant frequency in U-tube heat exchangers. The system integrates a piezoelectric sensing element embedded in protective encapsulation, signal-conditioning electronics, and a high-sampling-rate acquisition module. Experiments were conducted in a reduced-scale exchanger comprising 90 access ports and measurement depths up to 775 mm, using 28 kHz ultrasonic transducers. The probe successfully captured both the spectral content and the spatial variation of the voltage-based acoustic response along the tube bundle, revealing position-dependent amplitude variations and dominant-frequency measurements concentrated around the imposed excitation frequency. The analysis supported the definition of a reduced set of representative sampling locations, decreasing acquisition time while preserving the main spatial trends relevant to the objectives of this study. The procedures established here provide an experimental basis for future studies on the application of ultrasound to fouling-mitigation strategies in industrial thermal systems.

## 1. Introduction

The U-tube shell-and-tube heat exchangers consist of bundles of curved tubes fixed to a tube sheet, enabling fluid circulation through inlet and outlet manifolds. In industrial applications, particularly in offshore environments, these systems are prone to fouling due to the formation of inorganic deposits (e.g., calcium carbonate), biofilms, and other materials. Such fouling reduces the overall heat transfer coefficient, increases energy consumption, and compromises operational reliability. In this context, the development and optimization of cleaning strategies require experimental approaches capable of systematically reproducing fouling conditions under controlled environments.

Laboratory-scale models, such as the system employed in this study (internal volume of 0.018 m^3^ and tube inner diameter of 8 mm), provide a practical and efficient framework for this purpose. Reduced-scale configurations allow a large number of controlled experiments to be conducted with lower consumption of reagents and materials, which is particularly relevant when dealing with chemically induced scaling or biofilm formation. Although larger systems may better represent industrial conditions, they significantly increase operational complexity and resource demand. Consequently, laboratory-scale heat exchangers play a key role in the development and assessment of cleaning technologies, including ultrasonic cavitation. However, advancing these studies requires spatially resolved measurements of the acoustic field inside the tube bundle.

Accurate characterization of acoustic fields in confined liquid environments imposes strict constraints on sensor dimensions, mechanical integrity, and measurement fidelity. Conventional hydrophones provide high sensitivity and broad frequency response [1], but their use in small-diameter tubes is limited by probe size, mounting requirements, and vulnerability to intense cavitation. Cavitation processes generate localized high pressures, temperatures, and microjets capable of eroding solid materials [2,3], which can compromise fragile sensing elements. Even when miniaturized probes are available, extended operation under high-intensity cavitation and repeated axial scanning over long distances remain challenging.

Although the present work focuses on ultrasonic cavitation, related physical mechanisms also occur in hydrodynamic cavitation systems, such as Venturi-type reactors. In hydrodynamic cavitation, bubble formation is induced by local flow acceleration and pressure reduction at constrictions, whereas in ultrasonic cavitation it results from alternating acoustic compression and rarefaction cycles. Despite these different generation mechanisms, both processes involve bubble growth, collapse, localized energy concentration, microjet formation, shockwave emission, and strong near-wall effects. Recent studies on Venturi-type reactors [4] have shown that cavitation intensity and spatio-temporal behavior are governed by parameters such as cavitation number, Reynolds number, and thermodynamic effects, reinforcing the broader relevance of spatially resolved diagnostics for understanding cavitation-driven process intensification.

For this reason, many experimental studies rely on boundary-mounted sensors, external measurements, or point measurements in more accessible regions. These strategies are useful for monitoring global cavitation activity, but they do not provide direct information on the acoustic conditions inside narrow conduits. This limitation is particularly important because acoustic fields in confined geometries are inherently non-uniform. Previous studies have shown that broadband acoustic emissions associated with cavitation correlate with cleaning effectiveness [5], and that energy transport and cleaning performance in tubular systems depend strongly on distance from the transducer and cumulative sonication energy [6]. Other investigations have demonstrated that transducer arrangement, propagation distance, reflections, attenuation, and standing-wave effects strongly affect pressure distribution and cleaning efficiency [7,8,9]. From a practical perspective, this means that cleaning effectiveness in complex geometries may remain spatially heterogeneous, especially in confined or hard-to-reach regions [10].

Recent developments in acoustic sensing have addressed different parts of this problem. Hydrophone-based ultrasonic measurements have evolved from conventional piezoelectric devices, supported by well-established calibration and uncertainty procedures, to more specialized technologies for high-resolution, vector, optical, and cavitation-related measurements [11,12,13,14]. Fiber-optic microprobe hydrophones have provided very small sensing regions and high spatial resolution in cavitating or shockwave fields [15,16], while rugged miniature piezoelectric needle hydrophones have enabled direct measurements inside cavitation clusters, although cavitation damage to the sensing tip may still occur under severe conditions [17]. Commercial hydrophones combined with cavitation-monitoring systems have also been used to quantify cavitation noise and ultrasonic-field indicators in reactors and horn-based configurations [18,19].

More recent technologies include MEMS vector hydrophones for compact vector sensing and pipe-condition assessment [20,21], interferometric and fiber Bragg grating (FBG)-based optical hydrophones with high sensitivity and electromagnetic immunity [22,23], and engineered piezoelectric, composite, cymbal, flextensional, and PVDF hydrophones designed to improve bandwidth, sensitivity, waterproofing, or directional response [24,25,26,27,28]. In parallel, non-contact optical pressure-field imaging has been proposed to avoid hydrophone-induced field disturbance and spatial averaging [29], whereas hydrophone-validated numerical and diagnostic studies have contributed to reactor-scale acoustic-field prediction, cavitation characterization, and microbubble-spectrum analysis [30,31,32].

It should also be noted that high-resolution diagnostics in complex and confined fluid environments are not limited to acoustic sensing. Advanced techniques based on other physical principles, such as X-ray imaging and particle velocimetry, have been developed to investigate spatially resolved flow and density fields in optically inaccessible, multiphase, or porous media. These approaches address related challenges of spatial resolution and access in complex environments, although they measure different physical quantities and usually require different experimental infrastructure [33]. In contrast, the present work focuses on a miniaturized acoustic probe designed for direct insertion into small-diameter tubes and for mapping the ultrasonic response under confined heat-exchanger conditions.

Despite these advances, the available technologies are generally optimized for free-field metrology, underwater acoustics, biomedical ultrasound, low-frequency monitoring, optical-access configurations, or reactor-scale diagnostics. They do not usually combine, in a single device, sub-8 mm geometry, waterproof encapsulation, mechanical resilience under cavitation, compatibility with repeated axial insertion, and direct in situ mapping inside small-diameter U-tube bundles. Table 1 summarizes these sensing technologies according to the specific constraints of the present study.

Taken together, the literature indicates that, although several acoustic measurement technologies provide high sensitivity, broad bandwidth, optical resolution, vector information, or improved durability in specific applications, there remains a lack of compact and mechanically protected sensors designed for spatially resolved measurements inside small-diameter tubular geometries under cavitating ultrasonic fields. In particular, conventional, MEMS, optical, and commercial hydrophone systems are generally not configured for repeated axial insertion into 8 mm U-tubes while remaining fully submerged and mechanically protected against contact with tube walls and cavitation-related stresses. This limitation constrains experimental validation, hinders the interpretation of spatial cavitation patterns, and restricts the development of optimized ultrasonic cleaning strategies for confined heat-exchanger geometries.

The present study addresses this gap by developing and validating an experimental system for measuring relative acoustic response amplitude and dominant frequency inside confined water-filled tubes representative of shell-and-tube heat exchangers. The work includes the design of a miniaturized and mechanically reliable acoustic sensor compatible with 8 mm internal diameters, the development of an appropriate coupling and acquisition system, and the implementation of spatially resolved measurements along the tube axis. In addition, the influence of excitation conditions, particularly the number of active transducers, is evaluated, and reduced sampling strategies are assessed for efficient reduced-grid representation of the acoustic field.

## 2. Materials and Methods

In the present work, the measured acoustic response amplitude corresponds to the conditioned voltage output of the developed probe. Therefore, the spatial maps presented here should be interpreted as maps of relative voltage response resulting from the local ultrasonic field, rather than as absolute sound pressure distributions. Since neither a calibrated reference hydrophone nor a primary acoustic calibration system was available, the probe output was not converted into absolute pressure units. Nevertheless, relative amplitude mapping was sufficient for the objectives of this study, namely to compare spatial distribution patterns, identify regions of higher and lower response, and evaluate the influence of different transducer configurations while maintaining the same measurement chain and operating conditions. Thus, the term “acoustic response” refers to the conditioned voltage output from the developed probe under ultrasonic excitation; this signal is related to the local sound field but has not been calibrated in absolute terms of sound pressure and must, therefore, be interpreted as a relative amplitude of the acoustic signal.

**Figure 1:**
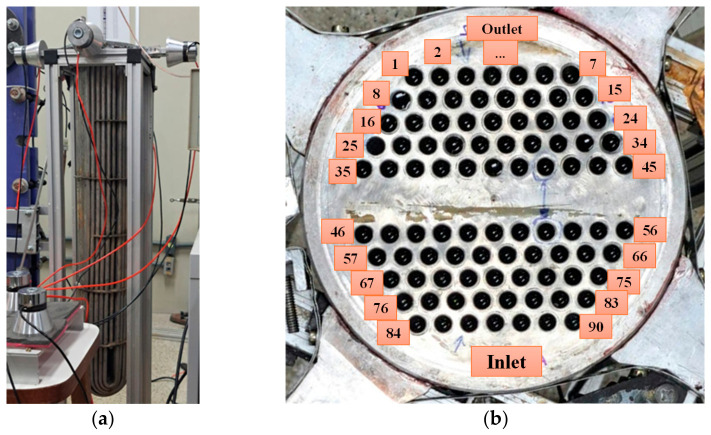
Tube bundle positioned vertically (**a**), externally to the shell, to enable mapping of acoustic response amplitude and frequencies through access via the tube sheet (**b**). Tube sheet indicating the inlet and outlet regions and the numbered measurement locations used for mapping.

The tube bundle used in the experiments consisted of 45 U-bend tubes, resulting in 90 access openings through the tube sheet, each with an internal diameter of 8 mm, as shown in Figure 1. This geometry reproduces, at reduced scale, typical conditions found in shell-and-tube heat exchangers used in offshore applications, allowing sensor insertion and acoustic evaluation along the tube depth.

Ultrasonic excitation was provided by 100 W Langevin transducers with a nominal operating frequency of 28 kHz, installed in diametrically opposite positions on the exchanger shell. Each transducer was coupled to the tube sheet through specially machined prolongation pieces whose front surfaces were shaped to match the local curvature of the tube sheet, thereby maximizing mechanical energy transfer from the emitting system to the tube bundle. Mechanical coupling was ensured by metallic compression straps and complemented by the use of Loctite 518^®^ anaerobic gel at the interface in order to reduce microgaps and improve transmission of ultrasonic vibrations.

**Figure 2:**
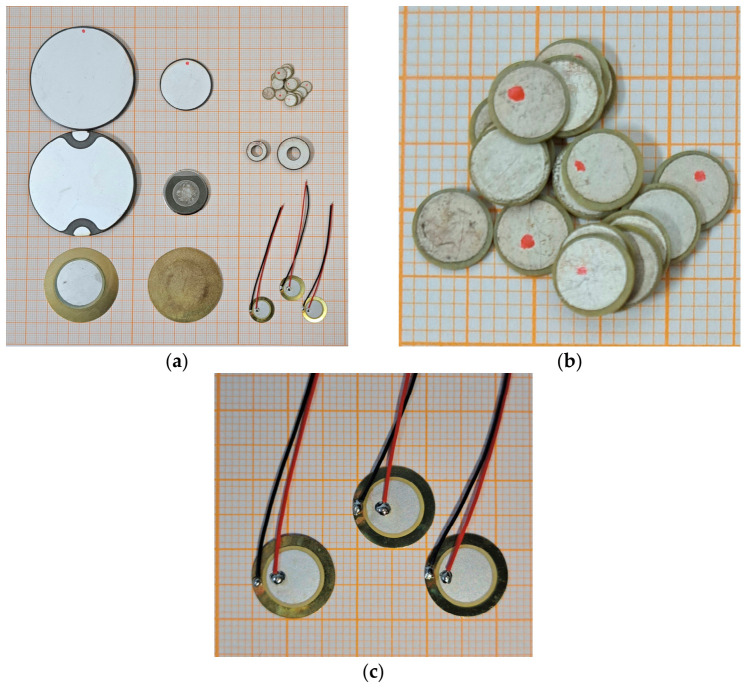
Commercial piezoelectric elements with different geometries and dimensions (**a**); Miniaturized piezoelectric elements evaluated during the sensor development (**b**); Flexible piezoelectric element selected for the proposed sensor. Dimensions are given in millimeters (**c**).

Figure 2 illustrates the broad range of commercially available piezoelectric elements tested during the exploratory phase of sensor development. The set shown in Figure 2a included elements with different diameters, thicknesses, and structural configurations, reflecting the large variety of geometries available for general ultrasonic applications. However, the miniaturized versions highlighted in Figure 2b showed unsatisfactory performance. Although their reduced dimensions were compatible with insertion into the bundle tubes, these elements exhibited unstable spectral response, low sensitivity, and intrinsic noise levels incompatible with the requirements of high-resolution acoustic mapping, thus compromising subsequent signal conditioning and acquisition.

By contrast, the piezoelectric elements shown in Figure 2c, flexible, low-cost components widely used in detection and ultrasonic excitation applications, displayed significantly better behavior. These components produced cleaner signals, lower electromechanical noise, and a more stable response in the frequency range of interest, allowing not only their integration into the acquisition system but also their geometric customization. The selection and modification criteria were based on four requirements: compatibility with insertion into 8 mm tubes, preservation of a sufficient active piezoelectric area, maintenance of electrical continuity at the soldered terminals, and mechanical robustness during encapsulation and handling.

To meet these requirements, the original piezoelectric element was geometrically reshaped by controlled mechanical trimming of the metallic support layer and peripheral regions. This procedure reduced the external dimensions of the element while preserving the active sensing region and enough metallic area for reliable soldering of the signal wires. The main technical challenge was the manipulation of very small and fragile components. Excessive material removal could reduce sensitivity, damage the active layer, compromise the electrode continuity, or weaken the soldering region. Therefore, the modification process required a compromise between miniaturization, signal stability, mechanical integrity, and electrical reliability before the element could be integrated into the final encapsulated probe.

**Figure 3:**
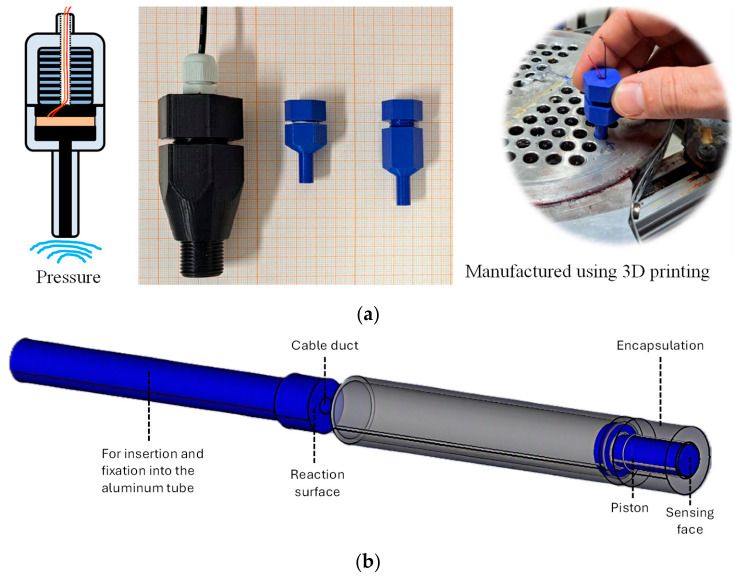
Piston-based system for momentum transfer and prototypes for near-surface measurements (**a**). Three-dimensional model of elongated housing (**b**).

The developed sensor was based on the principle of piston-like momentum transfer, in which the incident acoustic pressure acts at the end of a mechanical transducing element and transfers excitation to the piezoelectric element through confined axial displacements. As illustrated in Figure 3a, the measurand, in this case, the acoustic response amplitude field inside the tube bundle, was intercepted by a uniform cylindrical piston whose front face formed the hydrodynamic sensing surface. The opposite end of the piston was connected to an accommodation disk, responsible for transmitting the mechanical deformation to the piezoelectric element, which remained confined between this disk and an upper disk compressed by the threaded housing.

The assembly therefore operated as an axial mass–spring–damper system in which the piston acted as an intermediate element capable of:

(i) Ensuring geometric alignment between the measurement point and the piezoelectric element;
(ii) Preserving the integrity of the piezoelectric component by isolating it from lateral stresses and cavitation erosion;
(iii) Transmitting the axial component of the acoustic pressure with minimal attenuation.

A small lateral slot allowed the passage of flexible wires, which were routed through the rear portion of the housing without interfering with the free axial movement of the piston. The sensor geometry evolved iteratively through rapid prototyping, as indicated in Figure 3b. Several scales were tested, all produced by additive manufacturing in ABS, until an adequate combination of mechanical resilience, piezoelectric sensitivity, and hydraulic coupling quality was achieved. This stage was essential for adjusting tolerances, minimizing clearances that could introduce mechanical noise, and ensuring linearity in axial motion transfer.

Subsequently, a slender elongated housing was conceived to allow insertion of the sensor along the 8 mm tubes of the bundle, the external diameter of the sensor being 7 mm. The resulting three-dimensional model, shown in Figure 3b, fully preserved the piston-like operating principle described above while incorporating a narrow tubular body accommodating the piston, the piezoelectric element, and the wiring. This configuration ensured axial rigidity while minimizing disturbance to the local acoustic field. The elongated format was essential to enable deep measurements (approximately 725–775 mm) without significant lateral contact with the inner tube walls, thereby ensuring that the measured response remained representative of the acoustic field rather than of structural vibrations of the exchanger.

**Figure 4:**
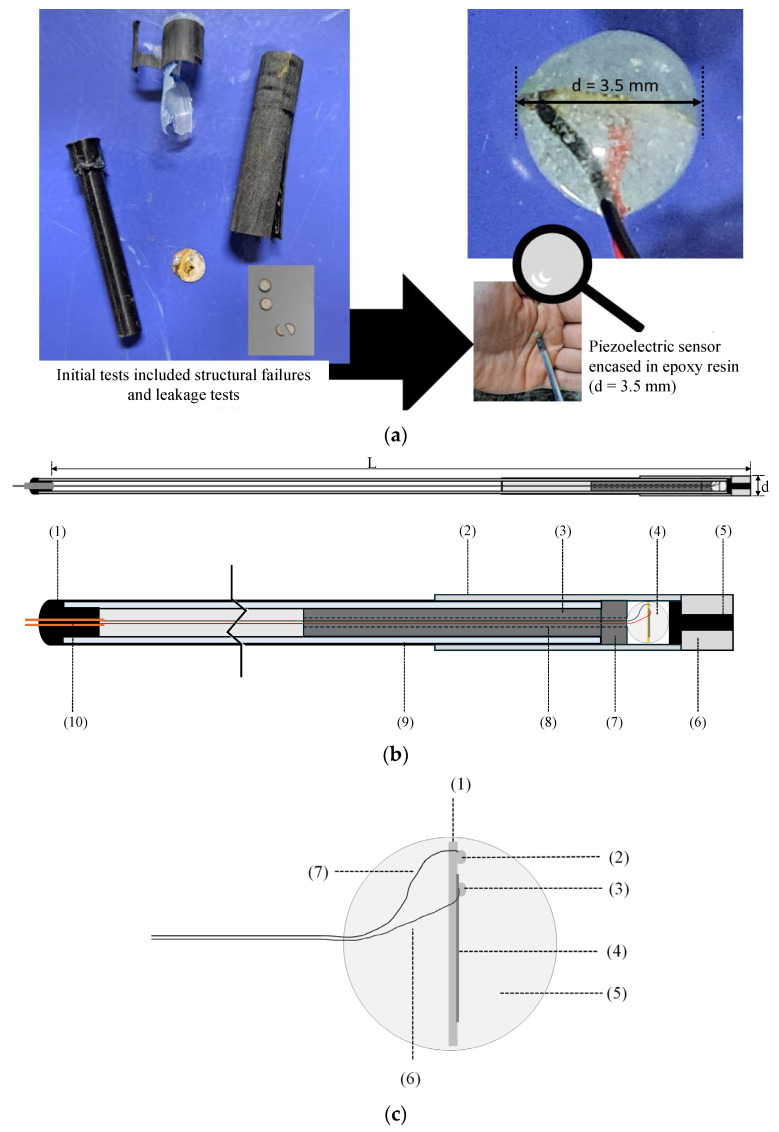
Initial tests and final version encapsulated in epoxy resin (**a**). Components of the developed instrumentation assembly (**b**): technical drawing indicating the useful length of the rod, L, and the diameter of the front part of the probe; Detail with: (1) cable gland; (2) lateral outer casing; (3) fastener; (4) resin encapsulation of the piezoelectric pellet; (5) piston; (6) front outer casing; (7) fixing head of the encapsulated pellet; (8) conduit for transmission cables; (9) tubular aluminum rod; (10) transition cabling. (**c**) Detail of the piezoelectric pellet encapsulation: (1) substrate of the piezoelectric material; (2) solder of the cabling (V−); (3) solder of the cabling (V+); (4) piezoelectric material deposited on the substrate; (5) epoxy resin encapsulation; (6,7) cabling encapsulated in epoxy resin.

Preliminary tests revealed important limitations in the first version of the piston-based sensor. The use of silicone as a sealing element between piston and housing proved inadequate to guarantee watertightness under prolonged immersion, resulting in leakage that compromised both the mechanical integrity and electrical stability of the system. At the same time, the rigid piezoelectric elements initially adopted (Figure 2b) were highly susceptible to rupture during insertion and removal of the sensor from the 8 mm tubes, as shown in Figure 4a. These failures were mainly associated with the limited space available for access, the low dimensional tolerance resulting from three-dimensional printing, and the significant lateral friction generated as the sensor was displaced along the tubes. These factors imposed undue mechanical stresses on both the piezoelectric element and the sealing region, leading to fracture, delamination, and loss of watertightness.

To overcome these limitations, an alternative solution was developed based on full encapsulation of the piezoelectric element in a low-shrinkage epoxy resin, resulting in an approximately spherical, mechanically robust, and hermetically sealed body. The final geometry, shown in Figure 4a–c, had an average diameter of 3.5 mm, sufficiently small to fit inside the piston-based housing without excessive friction while preserving acoustic sensitivity and eliminating the rupture and leakage issues observed previously. In addition to ensuring watertightness, the spherical encapsulation improved resistance to lateral impacts during displacement inside the tubes, significantly increasing the operational reliability of the system.

Although encapsulation of the piezoelectric element in epoxy resin was essential to ensure sealing and mechanical structural integrity, it added mass and modified the stiffness of the sensing assembly. These changes in the oscillating mechanical system caused significant attenuation of the captured acoustic signal amplitude when compared with the response obtained in preliminary tests performed with the non-encapsulated element. Owing to this loss of sensitivity, dedicated signal conditioning and amplification stage became necessary.

The conditioning stage was designed to increase the voltage level of the encapsulated sensor without intentionally modifying the temporal structure of the signal. No integration or low-pass filtering stage was introduced in the useful frequency range. The selected RC4558-based amplifier was operated within the 20–40 kHz band used in the experiments, and the gain was adjusted to avoid saturation of the output signal. Therefore, although the analog circuit may introduce small amplitude and phase changes inherent to any amplification stage, it was not expected to impose a relevant limitation on the response time required for the present measurements. This was verified experimentally by comparing the excitation frequency with the dominant frequency measured at the sensor output, which showed strong agreement over the tested range.

**Figure 5:**
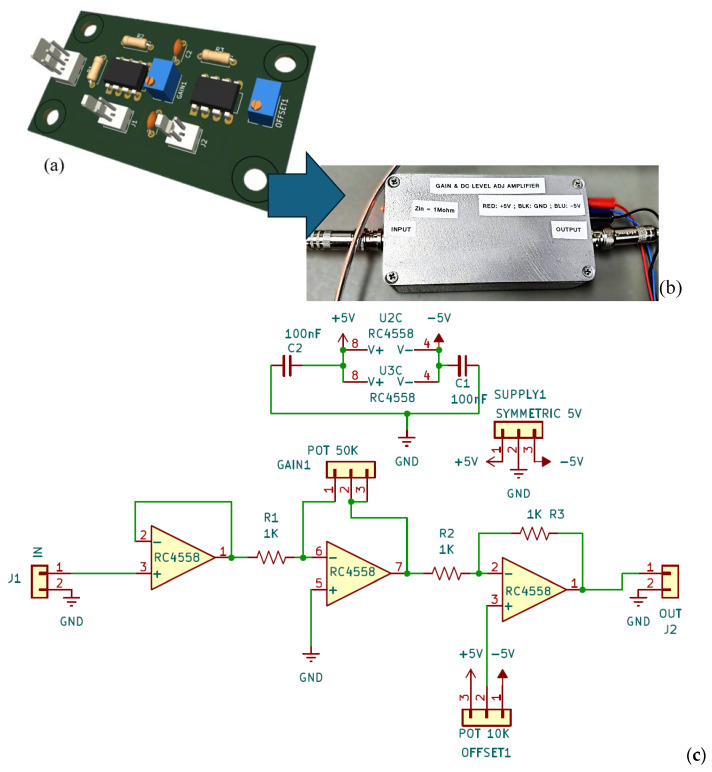
Signal-conditioning unit: (**a**) analog circuit CAD model; (**b**) aluminum enclosure assembly; (**c**) schematic of the piezoelectric sensor conditioner. A three-stage RC4558 circuit with adjustable gain and offset provides a 0–3 V output for the data acquisition system. The unit operates at ±5 V and includes electromagnetic shielding.

For this purpose, an analog circuit based on two RC4558 operational amplifiers (Texas Instruments, Dallas, TX, USA) [34] was designed to amplify the low-level sensor voltage signal (approximately 100 mV) to an adjustable output range between 0 and 3 V, as shown in Figure 5a. To reduce external electromagnetic interference, the signal conditioner was installed in a metallic Hammond-type aluminum enclosure, providing shielding and improving operational stability in the experimental environment (Figure 5b). Potentiometers integrated into the circuit enabled fine gain adjustment, allowing the system response to be adapted to the specific acoustic regime observed inside the tubes. The 50 kΩ gain adjustment potentiometer provides a maximum gain of 50, as the second stage was configured as an inverting amplifier [35]. The offset potentiometer was chosen with a typical value of 10 kΩ, and was used for DC level adjustment, establishing an offset of approximately 1.5 V. The circuit (Figure 5c) was powered by a symmetrical ±5 V supply to allow amplification of both positive and negative signal components. The RC4558 was selected because its gain–bandwidth characteristics are adequate for signals with high temporal variability. As a final circuit adjustment, a 1 MΩ resistor was added in parallel with the input BNC connector to improve impedance matching and signal transmission. This conditioning stage restored the usability of the encapsulated sensor by producing voltage levels compatible with the data acquisition system and enabling the subsequent testing and calibration stages.

The signal-conditioning circuit was implemented on a dedicated printed circuit board (PCB) designed for the sensor interface. The PCB layout was developed to accommodate the RC4558 operational amplifiers, gain and offset potentiometers, passive components, power-supply connections, and BNC-compatible input/output interfaces in a compact and mechanically stable arrangement. After PCB fabrication, the electronic components were assembled by manual soldering, followed by visual inspection of the solder joints and continuity verification before installation inside the aluminum enclosure. The potentiometers remained accessible for gain and offset adjustment during bench testing, whereas the metallic enclosure provided mechanical protection and electromagnetic shielding for the conditioning board.

The measurement path was evaluated at the system level rather than through an independent calibration of each electronic component. The main function of the conditioning circuit was to raise the low-level piezoelectric signal to the voltage range required by the acquisition system, while preserving the dominant frequency content and avoiding output saturation. During the experiments, the gain was manually adjusted so that the conditioned signal remained within the 0–3 V acquisition range, and the DC offset was set close to 1.5 V to allow both positive and negative fluctuations of the amplified waveform to be recorded.

The accuracy of the conditioned signal was controlled through three complementary procedures. First, the output waveform was continuously inspected with a digital oscilloscope to verify signal integrity, absence of clipping, and stable baseline level. Second, the acquisition chain was tested with a function generator and oscilloscope to verify temporal response and absence of aliasing within the useful operating band. Third, the dominant frequency obtained from the conditioned sensor signal was compared with the excitation frequency measured at the transducer input, showing strong agreement over the 20–40 kHz range.

Acoustic signal acquisition was carried out using an STM32F411 microcontroller (STMicroelectronics, Geneva, Switzerland), selected to provide a sampling rate compatible with the ultrasonic frequency range investigated in this study. The acquisition system was configured to operate at 800,000 samples per second (800 kS/s), corresponding to a Nyquist frequency of 400 kHz. This value is well above the 20–40 kHz frequency range used in the sensor validation tests and in the ultrasonic experiments, allowing reliable identification of the dominant frequency component and associated spectral content. Each acquisition window lasted 20 s, corresponding to approximately 16 × 10^6^ samples per measurement.

**Figure 6:**
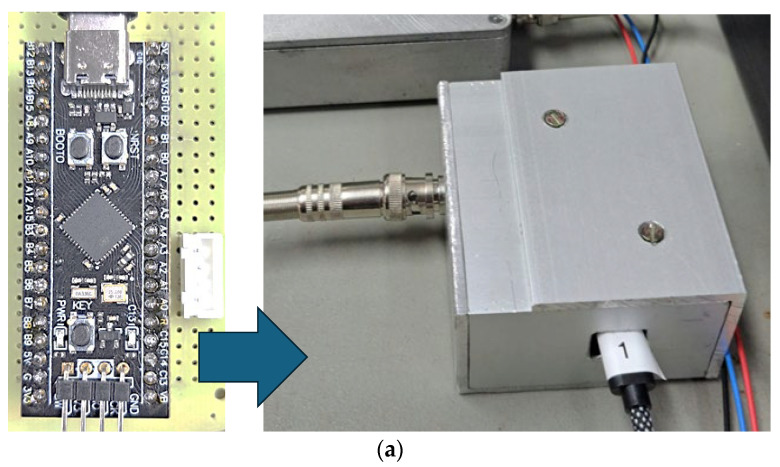

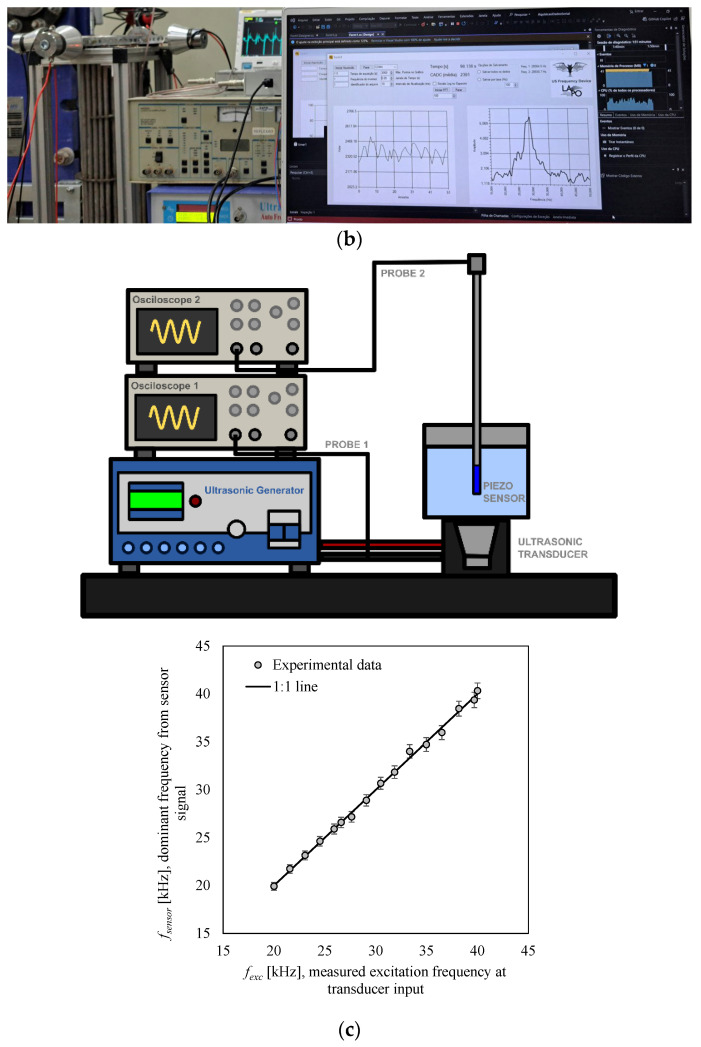
Microprocessor-based data acquisition system: (**a**) STM32F411 microcontroller and enclosure for shielding and cable connections; (**b**) data acquisition software interface with real-time FFT monitoring. (**c**) Comparison between excitation frequency measured at the transducer input and frequency obtained from the piezoelectric sensor, including the ideal 1:1 agreement line.

Communication with the computer was established via USB-C, whereas the conditioned signal was received through a BNC connector, ensuring electrical integrity and ease of decoupling among modules. The acquisition software stored the sampled data and provided FFT-based spectral visualization during the experiments. Therefore, the term “rapid data processing” refers here to the ability of the acquisition chain to sample and handle the sensor signal at 800 kS/s with real-time spectral monitoring, rather than to a separately measured processing-delay specification. To reduce external electromagnetic interference and improve operational stability, the microcontroller was mounted in a machined aluminum enclosure (Figure 6a).

To validate the frequency response of the developed sensor, an experimental test was conducted using a 100 W ultrasonic transducer coupled to a cylindrical aluminum reservoir filled with liquid. The sensor was positioned inside the system and subjected to controlled acoustic excitation in the range of 20–40 kHz. Two independent Tektronix TDS 2022C oscilloscopes (200 MHz bandwidth and 2GS/s) (Tektronix Inc., Beaverton, OR, USA) were employed: one connected directly to the transducer power supply cable to measure the excitation frequency, and the other connected to the sensor output to record the corresponding acoustic signal.

Figure 6c presents the comparison between the excitation frequency measured at the transducer input and the dominant frequency obtained from the piezoelectric sensor signal. The horizontal coordinates correspond to the actual frequency readings obtained during the tests, rather than to uniformly spaced nominal setpoints of the ultrasonic generator. In some cases, additional intermediate frequencies were also selected to further evaluate the sensor response within specific portions of the 20–40 kHz range. The results show strong agreement between both measurements, with a coefficient of determination R^2^ = 0.9977. The error bars represent the dispersion obtained from repeated acquisitions, with a maximum relative deviation of 2.04%, indicating good repeatability of the acquisition system. The small dispersion observed confirms that the sensor is capable of accurately tracking the dominant frequency component of the ultrasonic field within the investigated range, demonstrating its suitability for frequency measurements between 20 and 40 kHz.

**Figure 7:**
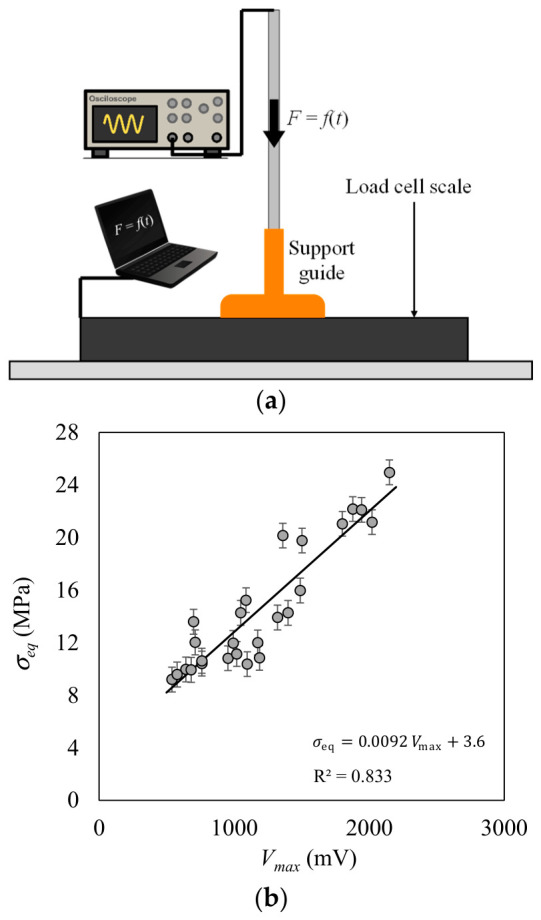
Experimental apparatus used for axial mechanical calibration of the sensor against a load-cell-based force-measuring plate (**a**), and equivalent normal stress applied to the sensor face as a function of the maximum sensor voltage response (**b**).

To relate the electrical response of the developed probe to the mechanical loading applied to its sensing region, a quasi-static equivalent mechanical calibration was performed using a load-cell-based force-measuring plate. The sensor was positioned inside a guide support and axially compressed against the plate, allowing the maximum applied force, *F*_max_, to be measured. The corresponding equivalent normal stress applied to the sensing face was calculated as:

(1) $$\sigma_{eq}=\frac{F_{max}}{A_{s}},$$

where $A_{s}=\pi d_{s}^{2}/4$ is the effective sensing area and $d_{s}=3.55\;mm$ is the measured diameter of the sensor tip. During each loading event, the sensor output voltage was recorded with an oscilloscope (Tektronix TDS 2022C; 200 MHz bandwidth) (Tektronix Inc., Beaverton, OR, USA), and the maximum voltage response was associated with the corresponding value of $\sigma_{eq}$. The resulting calibration curve, shown in Figure 7b, exhibited an approximately linear trend, described by Equation (2) with R^2^ = 0.833.

(2) $$\sigma_{eq}=0.0092\;V_{max}+3.6\;,$$

in which $\sigma_{eq}$ is the equivalent normal stress applied to the sensor face, in MPa, and $V_{max}$ is the maximum sensor voltage, in mV. The slope corresponds to an incremental equivalent mechanical response of approximately 108.7 mV/MPa, calculated as the inverse of 0.0092 MPa/mV. This value should be interpreted as the sensitivity of the complete sensor-conditioning chain to an equivalent quasi-static normal stress applied to the sensing face. It is not an absolute acoustic sensitivity in mV/Pa, because the calibration was performed under quasi-static mechanical loading rather than under a calibrated dynamic acoustic pressure field.

### 2.1. Operational Frequency-Response Characterization

A conventional acoustic calibration of the developed probe in terms of absolute sensitivity, expressed in V/Pa, was not available in the present study because no calibrated reference hydrophone or primary acoustic calibration system was available. Therefore, the acoustic signals reported in this work are not interpreted as absolute acoustic pressure values. The load-cell-based procedure described previously provides only a quasi-static equivalent mechanical calibration of the sensing face. To complement this characterization, an operational frequency-response test was performed to evaluate the dynamic response of the complete excitation–tank–fluid–probe–conditioning chain within the frequency range of interest.

**Figure 8:**
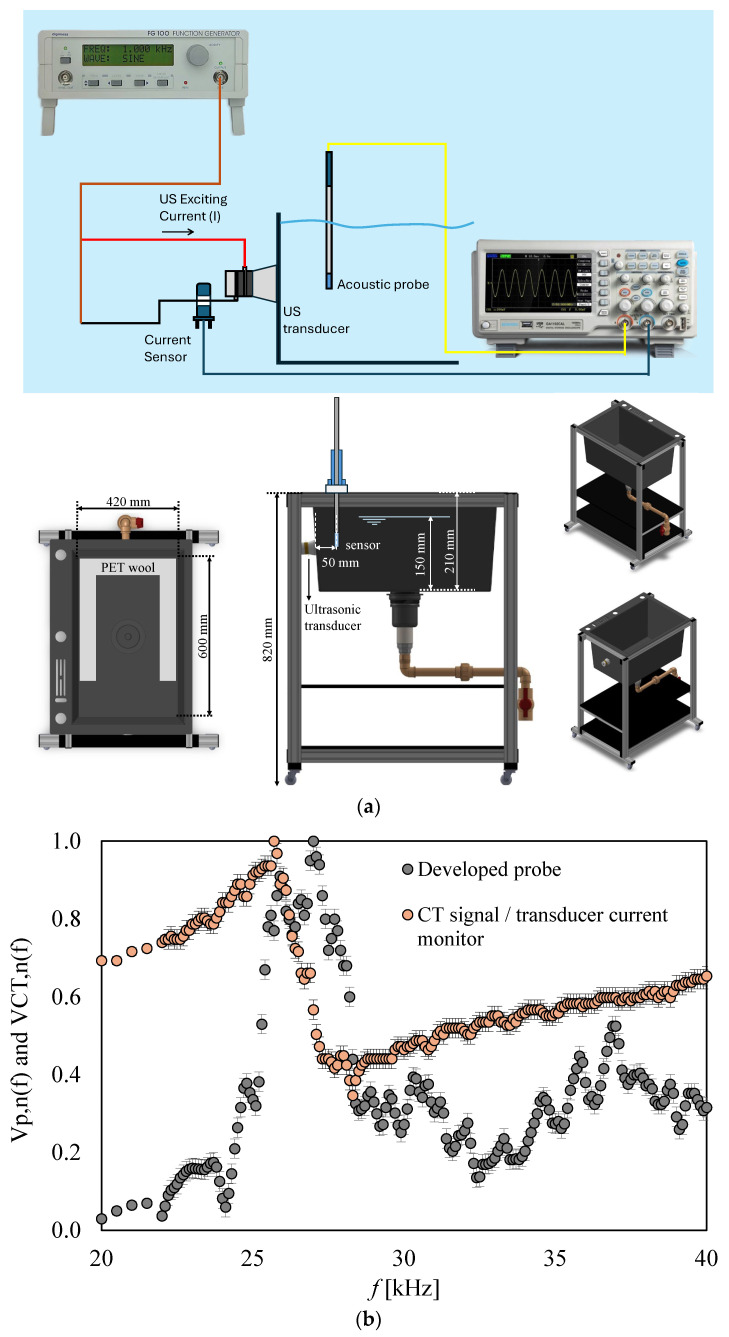

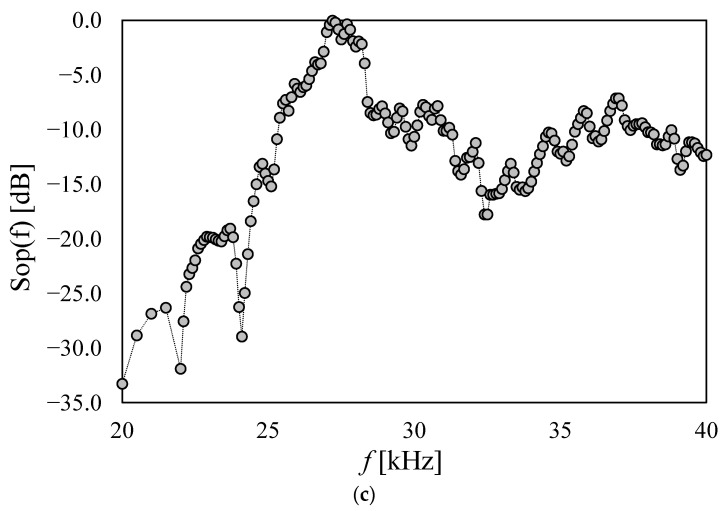
Operational frequency-response characterization of the developed probe. (**a**) Experimental setup with the water-filled tank, PET wool, 100 W ultrasonic transducer, and fixed probe position. (**b**) Normalized peak-to-peak voltages measured at the probe output and at the CT output used to monitor the transducer excitation current. (**c**) Normalized operational response, Sop, expressed in dB relative to its maximum value.

The test was carried out in a water-filled tank equipped with a 100 W ultrasonic transducer with nominal operating frequency of 28 kHz. The developed probe was kept fixed at a prescribed position inside the tank, as shown in Figure 8a. The transducer was excited by a sinusoidal function generator with an amplitude of 10 Vpp. This low-voltage excitation was selected to reduce nonlinear cavitation effects, excessive reflections, and waveform distortion, allowing a more stable evaluation of the frequency-dependent voltage response. PET wool was inserted into the tank to attenuate internal reflections and reduce the influence of standing-wave patterns during the sweep.

For each excitation frequency, two signals were recorded simultaneously using a digital oscilloscope. The first channel measured the peak-to-peak voltage at the output of the developed probe, Vpp,p(f). The second channel monitored the electrical excitation of the ultrasonic transducer using a current transformer, CT, installed around one of the transducer supply cables. The CT operates by electromagnetic induction: the alternating current flowing through the transducer cable produces a time-varying magnetic flux in the CT core, inducing an electrical signal in the secondary coil. Thus, the CT output voltage, Vpp,CT(f), was used as a relative signal proportional to the transducer excitation current. The 1 MΩ input impedance of the oscilloscope acted as the measurement load for the CT output. Both signals were first normalized by their respective maximum values over the tested frequency range:

Vp,n(f) = Vpp,p(f)/max[Vpp,p(f)](3)

VCT,n(f) = Vpp,CT(f)/max[Vpp,CT(f)](4)

where Vp,n(f) is the normalized probe-output voltage and VCT,n(f) is the normalized CT output voltage associated with the transducer excitation current. A relative operational response indicator, Rop(f), was then calculated as:

Rop(f) = Vp,n(f)/VCT,n(f)(5)

This ratio compensates, in relative terms, for frequency-dependent variations in the voltage applied to the transducer, while preserving the frequency-dependent response measured by the probe under the same experimental configuration. Finally, the normalized operational response in decibels was obtained as:

Sop(f) = 20log10{Rop(f)/max[Rop(f)]}(6a)

where Sop(f) is expressed in dB and has a maximum value of 0 dB. In the present dataset, max[Rop(f)] was equal to 2; therefore, this expression is equivalent to:

Sop(f) = 20log10[Rop(f)/2](6b)

This quantity should be interpreted as a relative operational voltage-response indicator, not as acoustic sensitivity in V/Pa. Since the acoustic pressure at the probe position was not independently measured, the test does not constitute an absolute hydrophone calibration. Nevertheless, it provides a reproducible characterization of the frequency-dependent behavior of the complete measurement chain under controlled small-signal ultrasonic excitation.

Figure 8 presents the operational frequency-response characterization of the developed probe. Figure 8b shows the normalized peak-to-peak voltages measured at the probe output and at the CT output used to monitor the transducer excitation current. The CT signal varied with frequency, indicating that the transducer excitation current was not perfectly uniform over the sweep. Therefore, the probe response was not evaluated directly from Vpp,p(f), but from the relative operational indicator Rop(f), calculated as the ratio between the normalized probe-output voltage and the normalized CT output voltage.

The resulting normalized operational response, shown in Figure 8c, exhibits a maximum near the nominal operating range of the ultrasonic transducer, approximately between 27 and 28 kHz. This behavior indicates that the developed probe and the complete measurement chain are most responsive in the frequency band used in the ultrasonic cleaning experiments. Away from this region, the response decreases, reaching values below −20 dB at the lower end of the tested range. The oscillations observed along the curve are attributed to residual modal behavior of the tank, incomplete suppression of reflections, coupling between the transducer and the tank wall, and the frequency-dependent response of the probe-conditioning system.

These results do not provide an absolute acoustic sensitivity in V/Pa. Instead, they provide a relative dynamic characterization of the developed probe under controlled small-signal excitation. Together with the quasi-static equivalent mechanical calibration and the frequency-tracking validation, this test supports the use of the probe for relative spatial mapping and dominant-frequency identification in confined ultrasonic fields.

The error bars shown in Figure 8b represent the instrumental voltage-reading uncertainty of the oscilloscope, taken as ±5 mV under the selected measurement scale. Since the plotted quantities are normalized voltages, this uncertainty was also normalized by the maximum value of each corresponding signal.

### 2.2. Data Acquisition in the Tube Bundle

The acquisition system was configured to operate at a sampling rate of 800,000 samples per second (800 kS/s), using 20 s acquisition windows. This provided adequate temporal resolution for spectral analysis of cavitation-related signals, as well as harmonics and subharmonics associated with the acoustic field generated by the transducers. A complete description of the acquisition system and its validation is presented by West et al. (2026) [36]. The acquisition and monitoring software was developed in C# and included parallel routines for:

- Continuous writing of data to .txt files in a dedicated thread;
- Real-time visualization of the frequency spectrum obtained through Fast Fourier Transform (FFT) of segmented sampling blocks, in analogy with the operation of digital oscilloscopes (Figure 6b).

**Figure 9:**
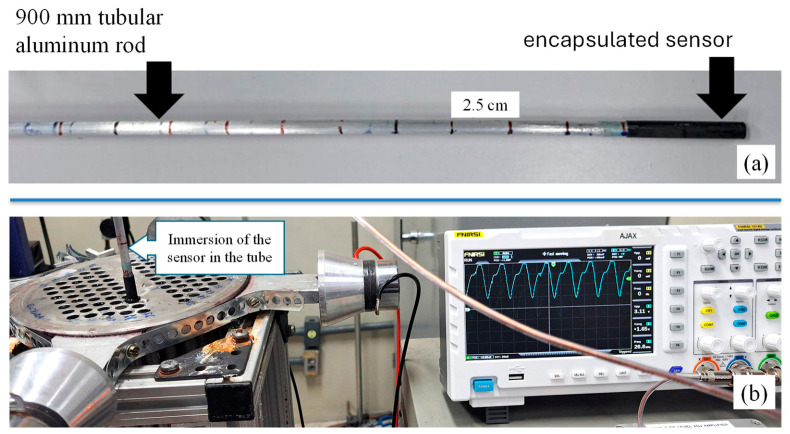
Sensor attached to a graduated aluminum rod (**a**); experimental setup with monitoring using an oscilloscope (**b**).

To validate the performance of both hardware and software, a Tektronix TDS 2022C oscilloscope (200 MHz, 2 GS/s) and a function generator were used to adjust gain, verify linearity, assess temporal response fidelity, and confirm the absence of aliasing within the useful operating band. The encapsulated sensor was attached to a rigid rod made from an aluminum tube through which the connection wires were routed to the upper end, thereby ensuring mechanical protection and positional stability during immersion. To allow controlled positioning along the axis of the exchanger tube, the rod was graduated at regular 2.5 cm intervals, enabling accurate definition of insertion depth at each measurement position (Figure 9a).

During the tests, acoustic signal behavior was monitored in two complementary ways. The first relied on the custom acquisition software, which provided real-time visualization of both waveforms and corresponding spectra. The second, adopted as metrological redundancy, used a digital oscilloscope connected directly to the output of the signal conditioner, allowing immediate verification of signal integrity, possible interference, and circuit saturation events (Figure 9b). This combined strategy improved the reliability of the measurements by facilitating identification of inconsistencies, cross-validation between instruments, and continuous monitoring of the dynamic behavior of the acoustic field at the investigated positions.

The experiments were initially conducted by performing measurements at four depths, 150, 300, 450, and 600 mm, in all 90 openings of the heat exchanger, with 20 s acquisition time per position. Given the adopted sampling rate, each measurement corresponded to approximately 16 × 10^6^ samples. In this preliminary stage, only one ultrasonic transducer was operated under a reference condition to map the general behavior of the acoustic field and identify regions of stronger response.

**Figure 10:**
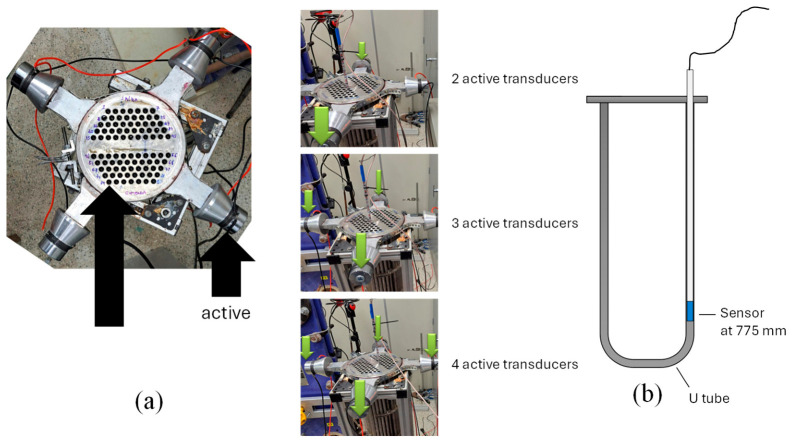
(**a**) Orifice No. 84 used in the preliminary test with 30 depth positions using a single transducer; (**b**) transducer arrangement for tests conducted at a depth of 775 mm.

Because a complete campaign covering all openings and depths required approximately 3.5 h of continuous operation, optimization of the number of sampling points per tube became necessary. This operational limitation was associated with the need for careful manipulation of the encapsulated sensor to avoid cable fatigue, resin failure, or lateral impacts during insertion. To guide this optimization, opening No. 84 (Figure 10a) was selected for a detailed study with 30 equally spaced depths, since exploratory tests identified this location as the one presenting the highest acoustic amplitude. It was therefore adopted as a representative case for defining a reduced sampling grid.

Additional tests were performed to compare different ultrasonic excitation configurations. At the maximum depth of 775 mm, the acoustic field was evaluated under the action of 2, 3, and 4 transducers (Figure 10b), allowing investigation of how the increase in the number of acoustic sources influenced the acoustic response distribution along the U-tube. For all experiments, the transducers were operated at 26.97 kHz, corresponding to the minimum electrical impedance condition. The external four-transducer bench was used throughout the experiments to ensure proper impedance distribution and reproducibility of the excitation arrangement. Temperature monitoring during all tests indicated stability at 34 °C, measured at the piezoelectric elements of the transducers.

## 3. Results

**Figure 11:**
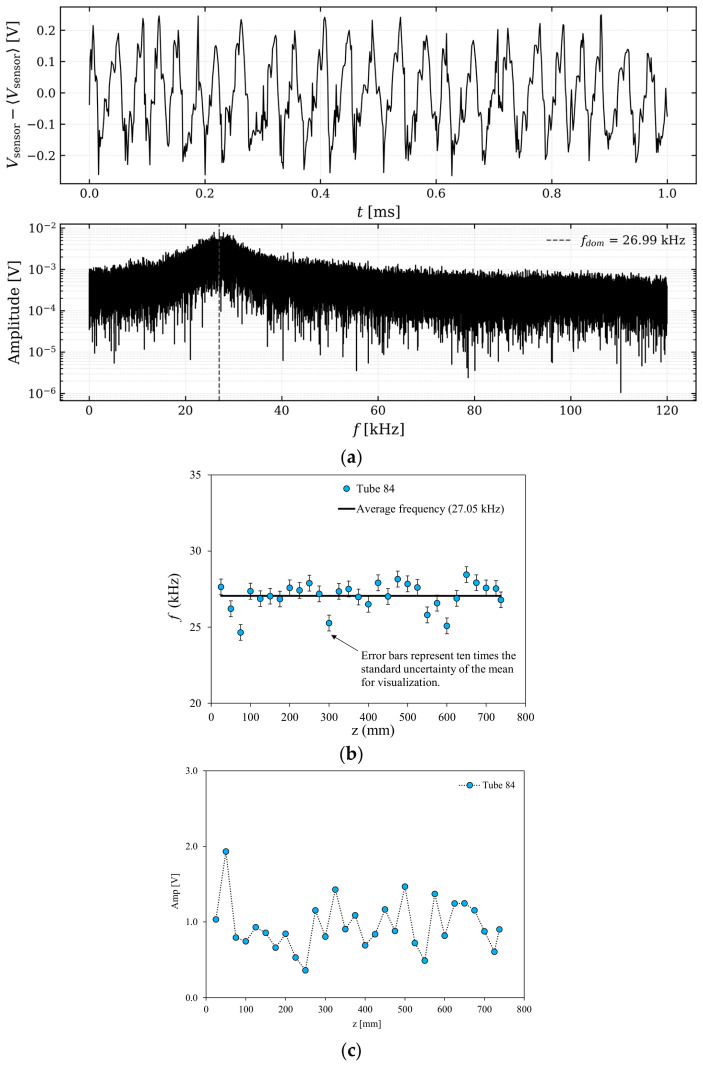

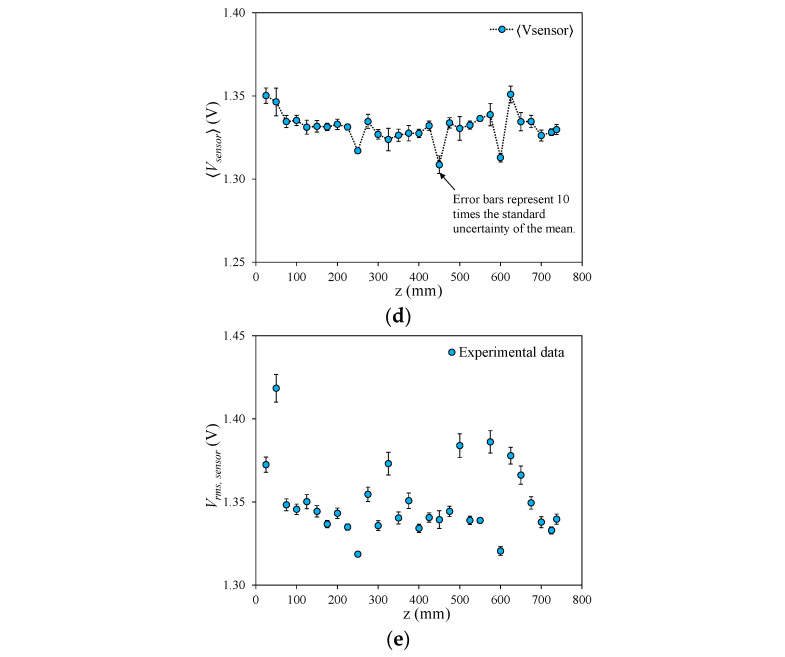
(**a**) Representative time-domain signal acquired in orifice 84 after DC-offset removal and corresponding FFT spectrum, with indication of the dominant frequency; distribution of frequencies (**b**) and amplitudes (**c**) along the depth of orifice 84 with a single transducer in operation; (**d**) Time-averaged sensor voltage, ⟨*V*_*sensor*_⟩, obtained from time-domain measurements over the acquisition window. (**e**) Distribution of the RMS voltage value along the depth.

Figure 11a presents a representative time-domain signal acquired in orifice 84 and its corresponding FFT spectrum. After removal of the DC offset for visualization, the signal shows a periodic component consistent with the imposed ultrasonic excitation. The spectrum exhibits a well-defined dominant peak at 26.99 kHz, in close agreement with the transducers’ operating frequency of 26.97 kHz, with an estimated signal-to-noise ratio of approximately 25.8 dB. This result confirms that the developed probe was able to capture the fundamental ultrasonic component with adequate spectral definition under the tube-bundle measurement conditions.

The analysis of the frequency distribution along the depth of orifice 84 indicates that the developed system exhibited stable and reproducible behavior. As shown in Figure 11b, the measured values are clustered around a mean frequency of 27.05 kHz, with a sample standard deviation of 0.91 kHz. This relatively small dispersion suggests that the sensor was able to capture the acoustic field within the tube, despite the complex geometry of the tube bundle and the variations in acoustic impedance associated with internal reflections. Furthermore, the close agreement between the measured mean frequency (27.05 kHz) and the excitation frequency imposed by the ultrasonic generator (26.97 kHz) indicates proper tuning between the transducer, its mechanical extension, and the acquisition system, reinforcing the reliability of the proposed instrumentation approach.

In contrast, the amplitude distribution, presented in Figure 11c, exhibits a markedly different behavior. A maximum value is observed near the tube inlet, at approximately z ≈ 50 mm; this is physically expected, as it represents a region of higher acoustic response amplitude (or acoustic intensity) associated with the proximity of the excitation source and lower accumulated attenuation. Beyond this point, the amplitude displays an oscillatory pattern along the depth, with a mean value of 0.91 V and a standard deviation of 0.28 V, indicating greater variability compared to the frequency measurements. This fluctuation results from the formation of interference patterns, including nodal and antinodal regions, typical of acoustic wave propagation in confined geometries. It also suggests the presence of quasi-standing acoustic fields arising from multiple reflections and geometric constraints, as well as local variations in cross-section and boundary conditions along the propagation path.

The distribution of the time-averaged sensor voltage, ⟨*V_sensor_*⟩, shown in Figure 11d, exhibits a relatively smooth spatial variation along the tube, with a mean value of 1.33 V and low dispersion, as indicated by the error bars derived from repeated measurements. This behavior indicates good stability of the conditioned signal baseline during the acquisition window. However, because this quantity includes the DC offset introduced by the signal-conditioning circuit, it should not be interpreted as a direct measure of acoustic energy. The RMS voltage shown in Figure 11e also remains nearly uniform along the tube, providing an additional indicator of signal-level stability during the measurements. Nevertheless, the spatial variation of the ultrasonic response is more directly represented by the amplitude distribution shown in Figure 11c, which exhibits an oscillatory behavior associated with attenuation, reflections, and interference within the confined U-tube geometry.

The higher amplitude observed near the tube sheet suggests that part of the incident ultrasonic energy is initially concentrated near the tube entrance before undergoing attenuation and redistribution along the propagation path. However, the subsequent oscillatory behavior indicates that the measured response cannot be interpreted as a simple monotonic decay from the nearest transducer. In the present configuration, the acoustic field results from a coupled fluid–structure problem: ultrasonic energy is introduced by external transducers attached to the tube sheet and shell, transmitted through the metallic structure, and then coupled into the water-filled U-tubes.

Under these conditions, each measurement point may receive contributions from different acoustic and structural paths, including waves transmitted through the tube sheet, waves propagating along the liquid column, and components reflected by the U-bend, tube entrances, neighboring tubes, and local geometric discontinuities. According to classical acoustic theory, the local response in a bounded domain depends on the superposition of these attenuated and phase-shifted contributions. As a result, constructive and destructive interference may produce local maxima and minima, giving rise to node-like and antinode-like regions along the tube.

This interpretation explains why the acoustic amplitude does not necessarily decrease monotonically with distance from the transducers and why high-response regions may occur in tubes that are not the closest to the excitation point. It also explains why the dominant frequency can remain close to the imposed excitation frequency, while the measured amplitude varies substantially with tube position and depth. Therefore, the spatial patterns observed in the present measurements suggest the occurrence of attenuated propagation, structural transmission, reflections, and interference within a confined U-tube bundle. A fully quantitative description of this field would require a three-dimensional coupled acoustic–elastic finite-element model including the tube sheet, shell, U-tube curvature, transducer coupling, and fluid domain, which is beyond the scope of the present experimental study.

For the tests conducted under the configurations shown in Figure 10b, with the sensor positioned at a depth of 775 mm, the mean acoustic signal amplitude between the two sides of the U-tube (B40–B51) was approximately 1.07 V when only two transducers were active. When three and four transducers were used, the mean amplitude increased to approximately 1.5 V. This increase indicates that the superposition of acoustic fields from multiple sources leads to a measurable amplification of the signal, even under conditions of significant attenuation along the tube length. These preliminary results demonstrate that the system response is sensitive to the number of active transducers, highlighting the importance of excitation configuration in the spatial distribution of ultrasonic energy.

**Figure 12:**
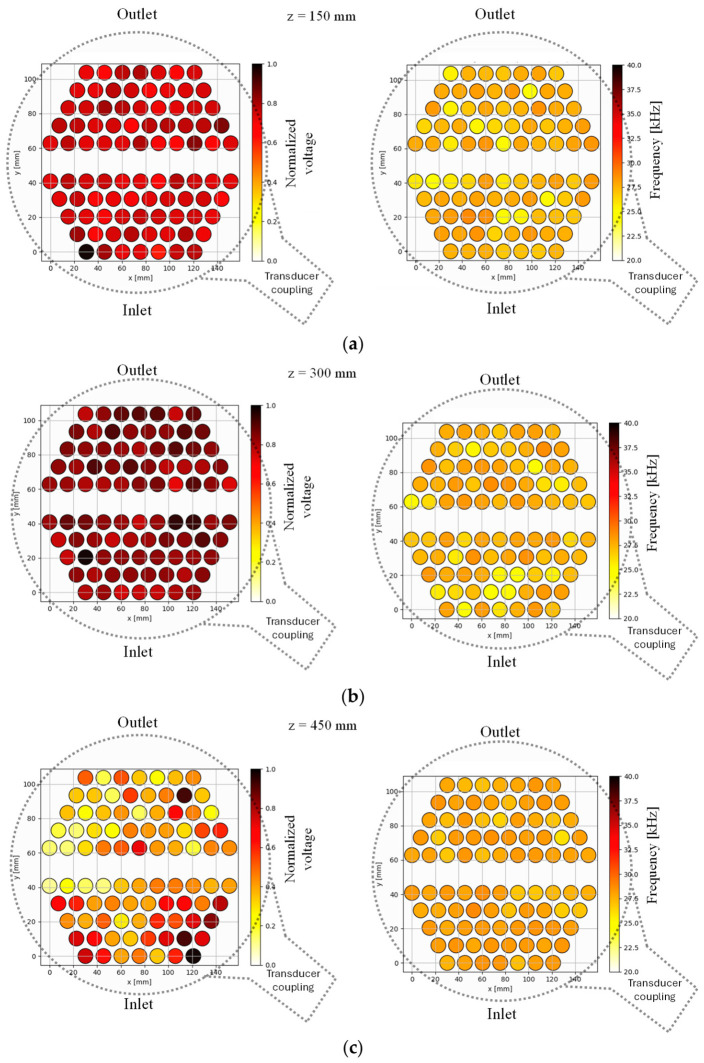

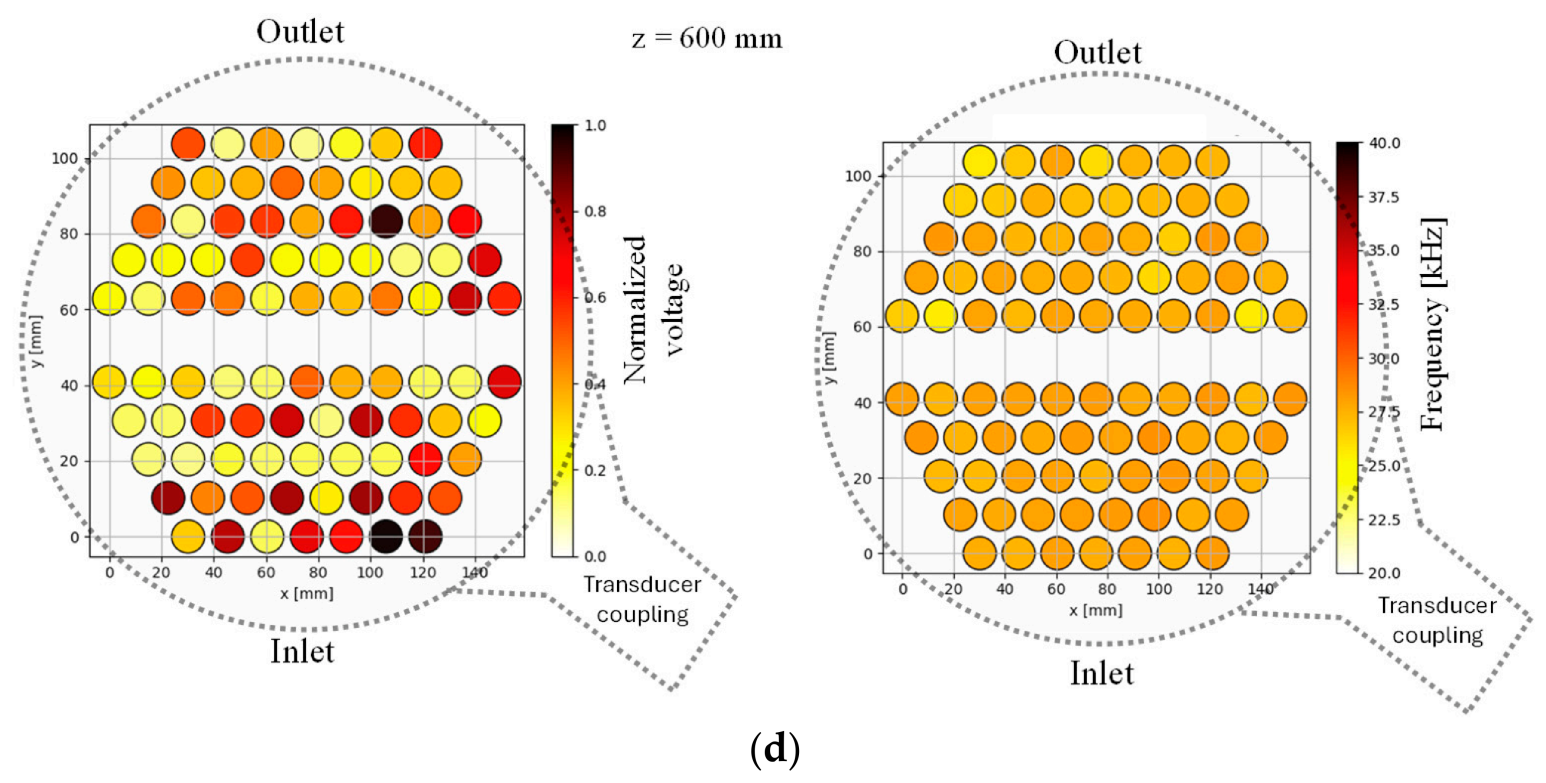
Mapping of the 90 orifices (U-tube sides) for depths up to 600 mm and 1 active transducer: (**a**) z = 150 mm; (**b**) z = 300 mm; (**c**) z = 450 mm; (**d**) z = 600 mm.

Following this stage, a complete mapping of the 90 openings of the tube bundle was performed, as described in the methodology, using a single transducer in operation to ensure repeatability and experimental control. The resulting maps, presented in Figure 12, show that the measured frequencies remained highly uniform across the entire bundle, with relatively small variations among tubes and increased stability at greater depths. This result suggests that the system operates predominantly under a single effective excitation mode at a frequency close to the imposed 26.97 kHz, which corresponds to the minimum electrical impedance condition of the transduction system.

The analysis of amplitudes, expressed as normalized voltage responses in the range [0, 1], reveals more pronounced spatial variations in the acoustic field. For shallow depths (z = 150 mm and z = 300 mm), a relatively uniform distribution across the tubes is observed, indicating that the acoustic field has not yet undergone significant attenuation and remains approximately homogeneous in the transverse plane. In contrast, at intermediate and greater depths (z = 450 mm and z = 600 mm), a higher degree of spatial dispersion is evident, with regions exhibiting reduced acoustic response amplitude. This trend is physically expected, given the progressive attenuation of the acoustic wave along the U-tube, as well as with internal reflections and interference phenomena associated with the curved geometry, which may locally amplify or attenuate the signal depending on position and phase relationships.

Overall, the results demonstrate that the developed system is capable of effectively mapping both the dominant frequency and the acoustic response amplitude along the tube bundle. The ability to identify regions of higher and lower acoustic response amplitude provides valuable insights for the optimized placement of transducers and for the design of ultrasonic cleaning strategies in shell-and-tube heat exchangers.

Figure 12 enables the visualization and analysis of the normalized acoustic response amplitudes, constituting an essential part of the proposed methodology for the appropriate selection of the transducer arrangement intended for the inspection and cleaning of shell-and-tube heat exchangers.

The average time of approximately 3.5 h required to measure all 90 openings at each depth demonstrated the operational impracticality of maintaining exhaustive mapping in all subsequent stages. Under these constraints, it became necessary to adopt a spatial sampling strategy grounded on the structure of the data obtained during the exploratory phase. The analysis presented in Figure 12 revealed a high degree of frequency uniformity, regardless of orifice location and depth, as well as a relatively stable spatial pattern for amplitudes, albeit with increased variability at greater depths. In situations where the physical field exhibits spatial continuity and low variation between adjacent points, the literature supports the use of reduced spatial sampling, provided that the selected points adequately represent the main gradient regions of the measured field [37]. This methodology increases experimental efficiency while preserving the ability to compare key spatial trends under a reduced-grid approximate sampling strategy. In addition to operational constraints, the adoption of a reduced sampling strategy was also motivated by the need to limit operator exposure to high-intensity ultrasonic noise, even under the use of hearing protection.

**Figure 13:**
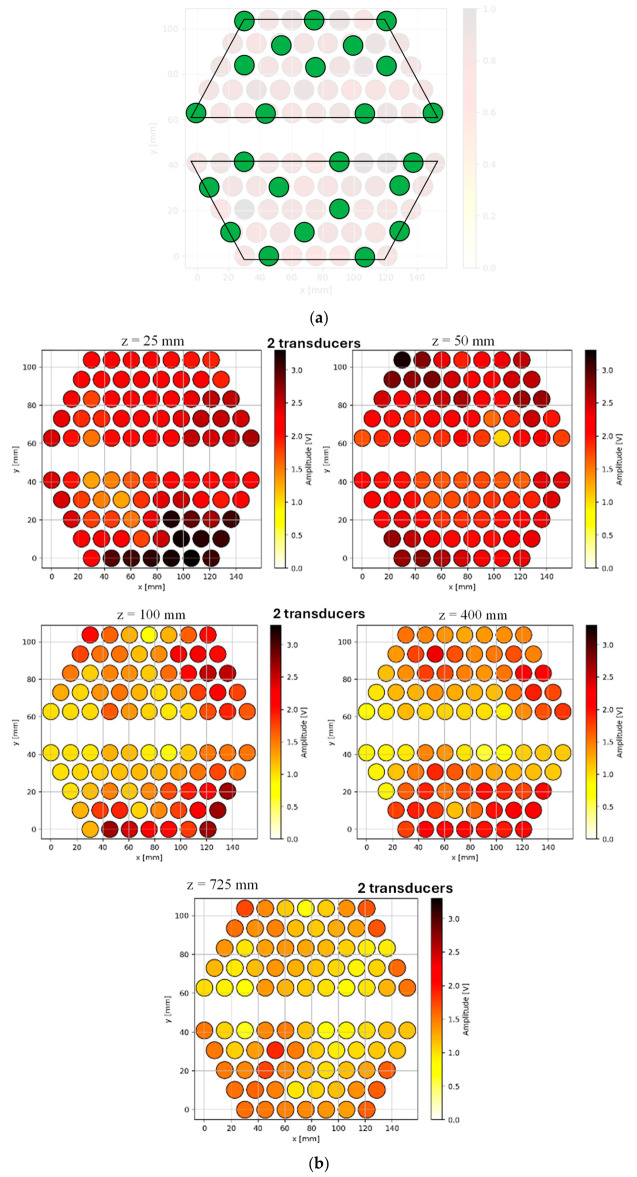

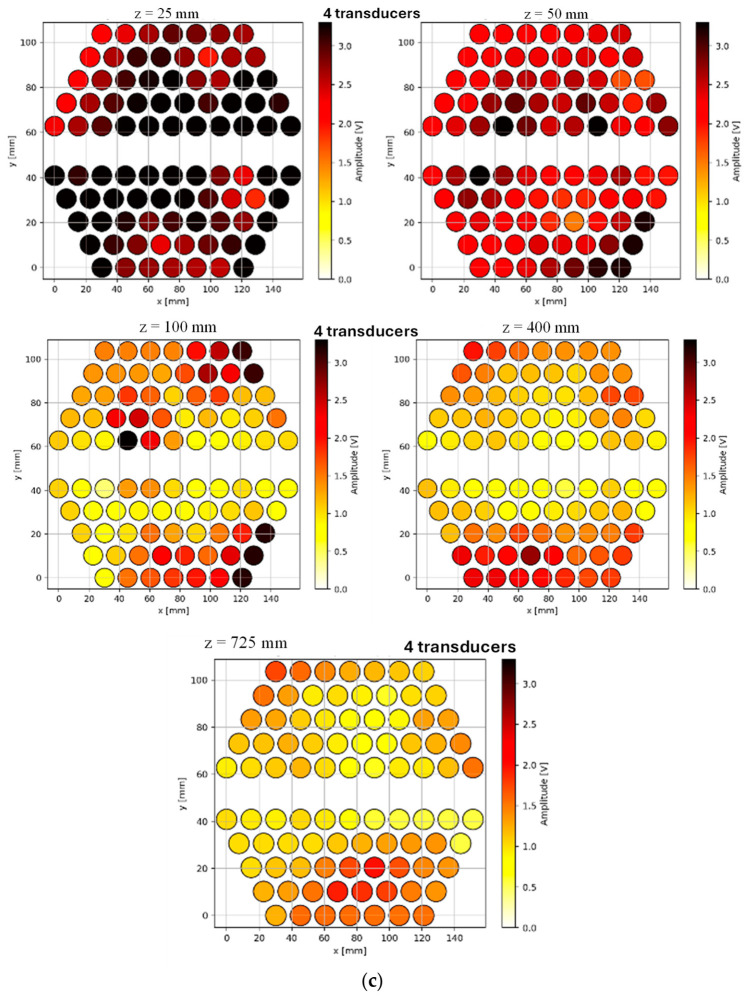
Sampling points based on previous results (green dots) (**a**) and reconstructed mapping using interpolation for two active transducers (**b**) and four active transducers (**c**).

Based on these principles, a reduced set of 24 sampling points was defined (Figure 13a), selected to capture the regions of greatest variability identified in the initial mapping while preserving the geometric characteristics of the tube bundle. This selection is in accordance with established methods for representative subset selection in spatial reconstruction, as discussed by [37], and with principles of efficient numerical discretization described by Press et al. [38]. Measurements were performed at depths of 25, 50, 100, 400, and 725 mm, under two operational configurations: two diametrically opposed transducers and four transducers operating simultaneously. From these measurements, the acoustic field was reconstructed using nearest-neighbor interpolation, a widely adopted method for reconstructing continuous fields from discrete samples [38].

The maps presented in Figure 13b,c should be interpreted as reduced-grid representations of the acoustic response obtained under the two- and four-transducer configurations, rather than as a direct reconstruction validation of the complete 90-aperture map. The complete mapping stage was performed with one active transducer and was used as an exploratory reference to identify the general spatial organization of the field and regions of higher variability. Based on this information, the 24-point grid was selected as a geometry- and response-informed sampling strategy, with points distributed to preserve coverage of the tube bundle while including representative regions near and far from the transducer-coupling zone. The observed spatial variations behaved like acoustic waves in confined geometries, where attenuation, reflections, modal interactions, and spatial redistribution depend on both the geometry and the excitation conditions [39,40]. Although a direct RMSE or correlation analysis against the 90-point map lacked physical significance, given the differing excitation conditions, the reduced strategy proved to be an adequate approximation for identifying the key spatial trends relevant to the study’s objectives.

The simplified grid was selected based on an analysis of the spatial pattern of the complete dataset, involving the choice of representative locations. This simplification strategy would require adjustments if there were changes to the number, location, or arrangement of the tube bundle. Such an approximation could be dispensed with if the data collection process were automated, for instance, through the use of robotic arms and automation strategies. In such scenarios, the fatigue and exposure experienced by the technician are replaced by the repetitive work of a machine, eliminating the need for tube sampling. Otherwise, an exploratory analysis will always be necessary to assess signal variability in the equipment under ultrasonic wave exposure.

**Figure 14:**
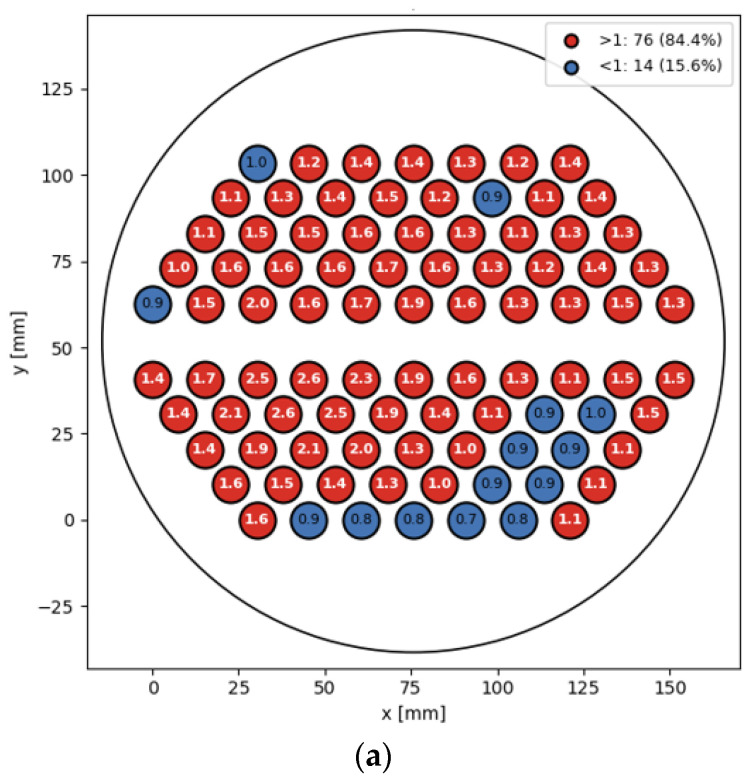

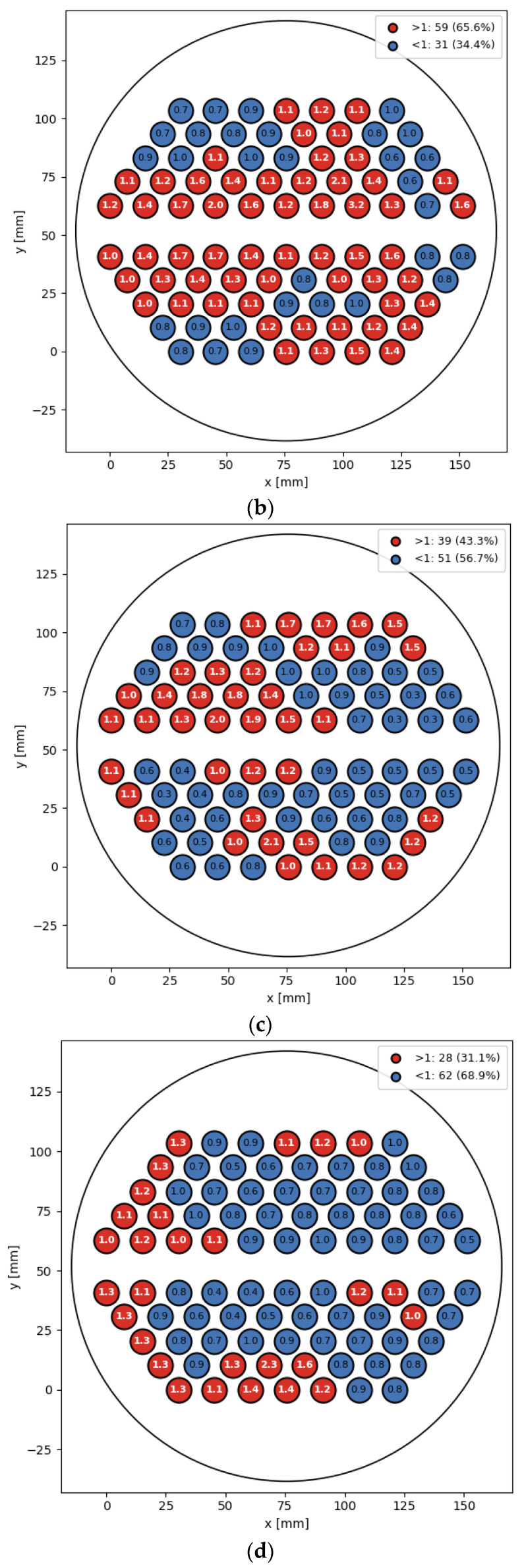

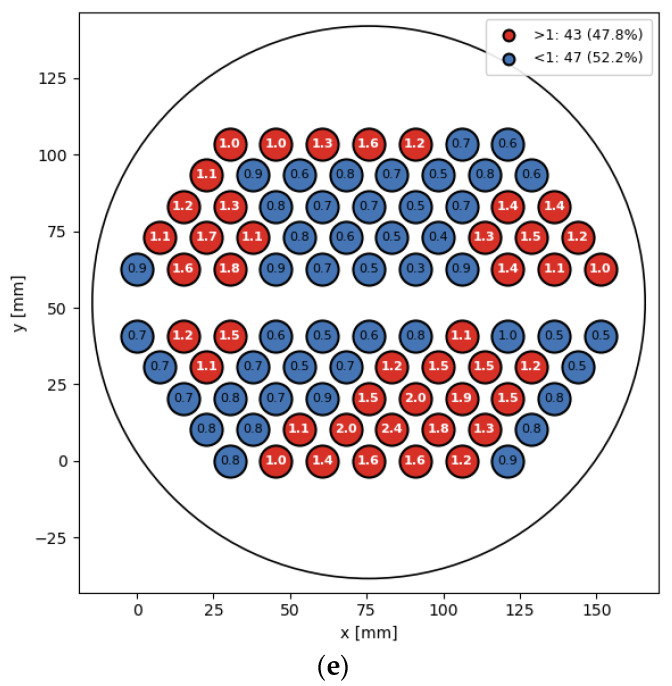
Performance index indicating the orifices and depths for which the acoustic response amplitude produced by four transducers exceeds that produced by two transducers (red), and where it is lower (blue): (**a**) z = 25 mm; (**b**) z = 50 mm; (**c**) z = 100 mm; (**d**) z = 400 mm; (**e**) z = 725 mm.

Interpolated maps allow for the proposition of different graphs for analyzing the efficiency of transducer combinations. In this work, the performance index x was defined as the ratio between the normalized acoustic response amplitudes obtained with four transducers and those obtained with two transducers. The results are summarized in Figure 14. At z = 25 mm, 84.4% of the orifices exhibited x > 1, indicating a clear gain associated with the use of a more spatially distributed acoustic energy source. At z = 50 mm, 65.6% of the orifices still showed improved performance with four transducers. However, at intermediate depths (z = 100 mm and z = 400 mm), a reversal in this pattern was observed, with only 43.3% and 31.1% of the orifices presenting x > 1, respectively. At z = 725 mm, the index increased again to 47.8%, reflecting the spatial oscillations previously identified in the full mapping.

These non-monotonic variations are physically expected for the case of ultrasonic wave propagation in long, confined ducts, where interference effects, local modal behavior, and differential attenuation can produce spatially complex patterns. In such systems, the superposition of multiple sources does not necessarily result in uniform amplification, but rather in position-dependent constructive and destructive interactions [17,18]. Nevertheless, even though acoustic response amplitude decreases in regions farther from the transducers, the working fluid in shell-and-tube heat exchangers is inherently forced to pass through the inlet and outlet regions, where the highest acoustic response amplitudes are consistently observed, thereby preserving the practical relevance of the proposed approach for fouling mitigation and cleaning processes.

The results obtained in the present study should also be interpreted in relation to the practical limitations of recently reported acoustic-pressure sensing technologies. Conventional hydrophones, fibre-optic microprobes, rugged needle hydrophones, MEMS-based devices, optical hydrophones, and engineered piezoelectric architectures provide important advances in sensitivity, bandwidth, spatial resolution, or signal stability. However, most of these systems are intended for free-field measurements, reactor-scale diagnostics, optical-access configurations, biomedical ultrasound, or underwater-acoustics applications. Their direct use inside small-diameter metallic U-tubes remains limited by probe size, fragility of the sensing tip, optical-access requirements, mounting constraints, or incompatibility with repeated axial insertion.

The developed probe addresses this application-specific gap by combining a 7 mm waterproof body, an epoxy-encapsulated sensing element, mechanical protection, and an elongated geometry suitable for axial displacement inside 8 mm tubes. This configuration enabled measurements at multiple depths and access openings of a U-tube heat-exchanger bundle, revealing the spatial variation of the ultrasonic response and the effect of different transducer configurations.

### Physical Interpretation of the Measured Acoustic Field

Although this work is predominantly experimental, the spatial distributions measured along the tubes can be qualitatively interpreted using a phenomenological model based on the superposition of attenuated harmonic wave contributions. In this framework, the complex acoustic-pressure-related field at a given position can be represented as [39]

(7) $$P\left(r\right)=\sum_{j=1}^{N}A_{j}\exp\left(-\alpha r_{j}\right)\exp\left[i\left(kr_{j}+\phi_{j}\right)\right]$$

where A_j_ is the amplitude associated with the j-th source contribution, r_j_ is the effective propagation distance from that source to the observation point, α is an effective attenuation coefficient, k is the acoustic wavenumber, and ϕ_j_ is the initial phase. In the present work, this expression is used only as a qualitative framework to interpret the measured voltage-response patterns. Since no absolute acoustic calibration was performed, the probe voltage should be interpreted as a relative indicator of the local acoustic response, rather than as an absolute measurement of acoustic pressure or intensity. Therefore, the measured local response depends on the coherent superposition of attenuated and phase-shifted wave contributions.

This expression predicts that increasing the number of transducers does not necessarily result in an increase in local acoustic response at all positions. Depending on the relative phases of the individual contributions, constructive interference may occur in some regions, while destructive interference occurs in others [39,40]. Furthermore, since acoustic propagation paths within the heat exchanger vary across tube rows and multiple reflections occur at the tube walls and U-bends, phase relationships vary with measurement depth, giving rise to complex spatial distributions such as those observed experimentally.

Although this formulation is not intended to provide a quantitative prediction of the acoustic field, it captures the essential physical mechanisms governing the measured spatial distributions.

Therefore, the non-monotonic evolution of the percentage of points exhibiting increased response amplitude is expected for an attenuated interference field propagating within a confined tubular structure [41,42]. The phenomenological model presented above provides a physically appropriate framework for interpreting the measured acoustic field distributions without requiring a full numerical solution of the acoustic wave equation.

Nevertheless, despite the progressive attenuation of the acoustic field as the distance from the transducers increases, the working fluid in shell-and-tube heat exchangers is inherently forced to pass through the inlet and outlet regions, where the highest acoustic response amplitudes are observed. Consequently, the proposed sensing approach remains highly relevant for monitoring acoustic fields associated with fouling mitigation and cleaning processes.

Absolute pressure calibration remains an important future step for quantitative use of the platform. In principle, the probe could be calibrated beforehand in a controlled acoustic field to determine its frequency-dependent sensitivity in mV/Pa, allowing conversion of the measured voltage response into an equivalent acoustic pressure under the calibration conditions. However, transferring this calibration to online or in situ measurements in confined cavitating fields is not straightforward. Here, online calibration refers to calibration or sensitivity verification with the probe installed and operating under conditions close to the actual measurement environment. In such conditions, the response may be affected by probe directivity, spatial averaging, wall proximity, reflections, fluid–structure coupling, nonlinear cavitation effects, and possible disturbance of the field by the inserted probe itself.

It is also important to distinguish the low-frequency ultrasonic excitation field from the broadband acoustic emissions associated with cavitation. The transducers used in this work generate a primary ultrasonic field in the tens-of-kHz range, which produces detectable wave components governed by propagation, reflection, interference, and structural coupling. By contrast, bubble oscillation and collapse can generate transient, damped, broadband acoustic emissions, including harmonics, subharmonics, ultraharmonics, and broadband noise components extending to much higher frequencies. The acoustic characterization of cavitation intensity is therefore not straightforward, since the measured spectrum may contain contributions from the direct ultrasonic field, nonlinear propagation, stable cavitation, transient cavitation, and sensor/system response [43].

In addition, cavitation behaviour and its acoustic signatures are affected by operating and boundary conditions, including liquid properties, local pressure, temperature, nuclei availability, wall proximity, and geometric confinement. In a related hydrodynamic-cavitation context, Ge et al. [44] showed that temperature-dependent thermodynamic effects can substantially modify cavitation intensity, cavity-flow structure, and regime transitions in Venturi-type reactors [44]. Although the present work deals with ultrasonic rather than hydrodynamic cavitation, this reinforces the broader point that cavitation fields are sensitive to external parameters and boundary conditions. Consequently, future online or in situ calibration of miniaturized probes in cavitating environments should define the target quantity to be monitored, whether the fundamental ultrasonic pressure field, harmonic components, or broadband cavitation-emission signatures, and should include frequency-dependent sensitivity, directional characterization, linearity assessment, bandwidth evaluation, and validation under representative confined geometries.

Recent studies have also shown that reduced-order methods, such as dynamic mode decomposition, can be used to identify coherent structures and classify cavitation regimes affected by thermodynamic effects [45]. Although the present work is limited to relative acoustic-response and dominant-frequency mapping, future studies could adapt such modal-decomposition and pattern-recognition tools to spatially distributed acoustic datasets to identify coherent ultrasonic/cavitation-field features and relate them to cleaning performance.

## 4. Conclusions

The development of the acoustic platform based on a miniaturized acoustic probe and its associated acquisition system enabled the experimental measurement of response amplitude and dominant frequency of the ultrasonic field inside a U-bend shell-and-tube heat exchanger with 8 mm internal diameter tubes. The sensor demonstrated performance compatible with depth-resolved mapping, allowing the acquisition of time series at sampling rates suitable for spectral analysis of ultrasonic signals.

Measurements conducted along the tube bundle showed that the dominant frequency remained stable across all evaluated positions, with variations consistent with the expected behavior of acoustic waves propagating in confined liquid media. In contrast, the amplitude exhibited clear spatial dependence, with higher values observed near the tube sheet region and progressive attenuation toward greater depths, in agreement with energy dissipation and geometric effects inherent to the system. The complete mapping of the 90 orifices revealed that the acoustic field is globally uniform in terms of dominant frequency, while the response amplitude displays spatial heterogeneity as a function of depth. The subsequent reduced-sampling approach proved to be a reasonable approximation of the patterns identified during the exploratory phase, demonstrating that a strategically selected subset of measurement points can significantly reduce experimental effort while preserving the representation of the main physical trends of the acoustic field.

Additional experiments with different excitation configurations showed that the use of four transducers produced higher response amplitudes in near-field regions, whereas at greater depths the results exhibited position-dependent variations associated with interference effects and spatial redistribution of acoustic energy. These findings indicate that the internal acoustic field within the tube bundle is sensitive to excitation conditions, particularly to the number and arrangement of active transducers, which should be considered in future ultrasonic applications aimed at fouling mitigation and cleaning.

Overall, the results demonstrate that the proposed experimental system can characterize confined ultrasonic acoustic fields in small-diameter tubular geometries, providing spatially resolved information on response amplitude and dominant frequency. The developed approach constitutes a reliable tool for investigating ultrasonic wave propagation in confined environments and offers a solid basis for future studies on the interaction between acoustic fields, cavitation phenomena, and fouling processes in heat exchangers.

The phenomenological interpretation proposed for the experimentally observed spatial distributions is consistent with the expected behavior of attenuated harmonic waves propagating in confined tubular structures, providing a simple physical framework for qualitatively understanding the measured interference patterns without the need for a full numerical acoustic model.

Future work can extend the application of the developed probe to heat exchangers with different tube diameters, tube arrangements, and access conditions in order to evaluate the adaptability of the design to other confined geometries. Additional studies should also investigate the spectral response of the acoustic field under different excitation frequencies, transducer arrangements, and power levels. In this context, the probe may be used not only as a diagnostic tool for spatial mapping, but also as a feedback element for the development of adaptive ultrasonic descaling systems, in which transducer operation could be adjusted according to the measured acoustic response inside the equipment. More broadly, the piston-like sensing concept, waterproof epoxy encapsulation, and elongated miniaturized geometry may be adapted to other applications requiring robust acoustic probes for confined or harsh liquid environments. Future metrological work should include absolute acoustic calibration, directional characterization, and long-term durability tests to further support quantitative use of the probe.

Regarding the industrial implementation of the developed probe, the following aspects should be considered: studying the stability of the various materials used—in terms of their chemical characteristics and interaction with industrial fluids—and implementing automated systems to move the probe between different access ports, thereby reducing human exposure to risks such as ergonomic hazards and toxicity inherent to industrial environments. Additionally, probes could be designed for multi-channel data acquisition, or robotic probes could be developed for remote data collection and transmission to enable internal monitoring—though this presents a significant challenge due to the harsh physical and chemical conditions within a heat exchanger, as well as the interference and localized pressure drop the probe might induce. Finally, probe data could be correlated with fluid–structure interaction simulation results to provide a comparative and complementary assessment of the system’s acoustic response under various excitation conditions.

## Figures and Tables

**Table 1:** Representative hydrophone and acoustic-pressure sensing technologies relevant to ultrasonic-field measurements, emphasizing their limitations for direct in situ mapping inside small-diameter U-tube bundles.

Sensing Technology	Representative Works	Demonstrated Capability	Remaining Gap for Confined U-Tube Measurements
Conventional piezoelectric hydrophones	Harris et al.; Isaev [13,14]	Established ultrasonic metrology, calibration procedures, broad frequency coverage, and well-defined hydrophone classes	Not designed for repeated axial insertion into 8 mm U-tubes under cavitation and mechanical contact
Fibre-optic microprobe hydrophones	Koch and Jenderka; Lokar et al. [15,16]	Very small sensing tips, high spatial resolution, and measurements in cavitating or shockwave fields	Require optical interrogation and precise alignment; not optimized as mechanically protected probes for long axial scans in industrial tubes
Rugged miniature piezoelectric needle hydrophones	Koch [17]	Small, broadband, purpose-made hydrophones for direct measurements inside cavitation clusters	Cavitation damage and repair of the sensing tip were reported; long-depth scanning in U-tube bundles was not addressed
Commercial hydrophones for cavitation monitoring	Bandelin et al.; Soyama et al. [18,19]	Practical detection of cavitation noise and ultrasonic-field indicators in reactors or horn-based systems	Sensor diameter and mounting configuration are not compatible with 8 mm tube insertion and internal tube-bundle mapping
MEMS vector hydrophones	Zhang et al.; Shi et al. [20,21]	Miniaturized vector sensing and simultaneous pressure/particle-velocity measurements, including pipe applications	Observed primarily at low frequencies and on larger piping scales
Interferometric and FBG optical hydrophones	Wang et al.; Chang et al. [22,23]	High sensitivity, electromagnetic immunity, and directional or interferometric acoustic detection	Generally low-frequency and/or centimeter-scale architectures; optical complexity limits use in narrow opaque tubes
Piezoelectric vector, composite, cymbal, flextensional, and PVDF hydrophones	Roh et al.; Je et al.; Kim and Roh; Kim et al.; Hou et al. [24,25,26,27,28]	Improved sensitivity, bandwidth, waterproofing, directional response, or low-frequency performance	Macroscopic, planar, diaphragm, or mechanically amplified geometries are not compatible with repeated insertion into 8 mm U-tubes
Non-contact optical pressure-field imaging	Ichihara et al. [29]	High-resolution spatiotemporal pressure imaging without hydrophone-induced field disturbance	Requires optical access and is unsuitable for opaque metallic tube bundles or internal measurements in 8 mm tubes
Hydrophone-validated reactor and cavitation diagnostics	Zhu et al.; Gong et al.; Liu et al. [30,31,32]	Acoustic-field validation, cavitation diagnostics, microbubble characterization, and reactor-scale ultrasonic modeling	Focus on reactors, transducers, cavitation mechanisms, or inverse diagnostics rather than a dedicated insertable sensor for U-tube mapping
Present study	This work	Waterproof 7 mm piezoelectric probe for in situ axial mapping inside 8 mm U-tubes, with reduced spatial sampling for practical field representation	Provides relative/equivalent acoustic-field mapping

## Data Availability

Data is contained within the article.

## Abbreviations

The following abbreviations are used in this manuscript:

MEMS	Micro-Electro-Mechanical Systems
ABS	Acrylonitrile Butadiene Styrene
BNC	Bayonet Neill-Concelman
FFT	Fast Fourier Transform
RMS	Root Mean Square
CT	Current Transformer
FBG	Fiber Bragg Grating
PVDF	Polyvinylidene Fluoride
PET	Polyethylene Terephthalate

## References

[1] Preston R.C. (1986). Measurement and characterisation of the acoustic output of medical ultrasonic equipment Part 1. Med. Biol. Eng. Comput..

[2] Duck F.A. (2007). Medical and non-medical protection standards for ultrasound and infrasound. Prog. Biophys. Mol. Biol..

[3] Pflieger R., Nikitenko S.I., Cairós C., Mettin R. (2019). Characterization of Cavitation Bubbles and Sonoluminescence.

[4] Ge M., Sun C., Zhang G., Coutier-Delgosha O., Fan D. (2022). Combined suppression effects on hydrodynamic cavitation performance in Venturi-type reactor for process intensification. Ultrason. Sonochem..

[5] Uchida T. (2021). Quantitative evaluation of ultrasonic cleaning ability using acoustic cavitation signal. Jpn. J. Appl. Phys..

[6] Järvinen M., Ahmadzai S., Rauhala T., Moilanen P., Zettler H.U., Ishiyama E.M. (2024). Understanding the delivery of ultrasonic cleaning effect in a heat exchanger tube. Proceedings of the International Conference on Heat Exchanger Fouling and Cleaning 2024, Lisbon, Portugal, 21–26 April 2024.

[7] Wei L., Liu S., Dong F. (2025). Investigation into Enhancing Ultrasonic Cleaning Efficiency Through Symmetrical Transducer Configuration. Symmetry.

[8] Wang Y., Gao C., Yao G., Shang Y. (2026). Experimental study on ultrasonic wave propagation and attenuation characteristics in heat exchangers. Therm. Sci..

[9] Zhu X., Das R.S., Bhavya M.L., Garcia-Vaquero M., Tiwari B.K. (2024). Acoustic cavitation for agri-food applications: Mechanism of action, design of new systems, challenges and strategies for scale-up. Ultrason. Sonochem..

[10] Murcek R., Finster M., Georgi A.T., Prabhu S., Fuchs E., Mauermann M., Zettler H.U., Ishiyama E.M. (2024). Development of novel cleaning systems for improving immersion cleaning based on the hunting behaviour of the pistol shrimp. Proceedings of the International Conference on Heat Exchanger Fouling and Cleaning 2024, Lisbon, Portugal, 21–26 April 2024.

[11] Georgiou O., Frier W., Freeman E., Pacchierotti C., Hoshi T. (2022). Ultrasound Mid-Air Haptics for Touchless Interfaces.

[12] Choi S., Lee H., Moon W. (2010). A micro-machined piezoelectric hydrophone with hydrostatically balanced air backing. Sens. Actuators A Phys..

[13] Harris G.R., Howard S., Hurrell A., Lewin P.A., Schafer M.E., Wear K.A., Wilkens V., Zeqiri B. (2023). Hydrophone measurements for biomedical ultrasound applications: A review. IEEE Trans. Ultrason. Ferroelectr. Freq. Control.

[14] Isaev A.E. (2025). Determination of the hydrophone phase response during periodic calibrations. Meas. Tech..

[15] Koch C., Jenderka K.-V. (2008). Measurement of sound field in cavitating media by an optical fibre-tip hydrophone. Ultrason. Sonochem..

[16] Lokar Ž., Petelin J., Horvat D., Agrež V., Petkovšek R. (2026). Laser induced cavitation bubble and shockwave measurements with fiber optical hydrophone very close to origin. Exp. Therm. Fluid Sci..

[17] Koch C. (2016). Sound field measurement in a double layer cavitation cluster by rugged miniature needle hydrophones. Ultrason. Sonochem..

[18] Bandelin J., Lippert T., Drewes J.E., Koch K. (2018). Cavitation field analysis for an increased efficiency of ultrasonic sludge pre-treatment using a novel hydrophone system. Ultrason. Sonochem..

[19] Soyama H., Ueda R., Kajiwara K., Yashiro W., Yeo S.-H. A hemispherical bubble induced by ultrasonic vibration observed by high-speed X-ray imaging.

[20] Zhang G., Liu M., Shen N., Wang X., Zhang W. (2017). The development of the differential MEMS vector hydrophone. Sensors.

[21] Shi P., Yu Y., Watts J., Zhang G., Horoshenkov K.V. (2025). An application of a MEMS vector hydrophone for condition assessment of a water supply pipe. Appl. Acoust..

[22] Wang W., Pei Y., Ye L., Song K. (2020). High-sensitivity cuboid interferometric fiber-optic hydrophone based on planar rectangular film sensing. Sensors.

[23] Chang I.-N., Li W.-C., Kuo C.-C., Liu W.-F. (2026). A novel two-dimensional hydrophone based on fiber Bragg gratings. Sensors.

[24] Roh T., Yeo H.G., Joh C., Roh Y., Kim K., Seo H.-S., Choi H. (2022). Fabrication and underwater testing of a vector hydrophone comprising a triaxial piezoelectric accelerometer and spherical hydrophone. Sensors.

[25] Je Y., Sim M., Cho Y., Lee S.-G., Seo H.-S. (2023). Theoretical and experimental studies on sensitivity and bandwidth of thickness-mode driving hydrophone utilizing a 2-2 piezoelectric single crystal composite. Sensors.

[26] Kim D., Roh Y. (2023). Design and fabrication of a high-sensitivity and wideband cymbal hydrophone. Sensors.

[27] Kim G., Kim D., Roh Y. (2024). Design of wideband flextensional hydrophone. Sensors.

[28] Hou T., Piao Z., Wang Y., Xin Y. (2026). A biomimetic tympanic cavity PVDF hydrophone for low-frequency bioacoustic monitoring in marine aquaculture. Sensors.

[29] Ichihara S., Yamagishi M., Kurashina Y., Ota M., Tagawa Y. (2025). High-resolution pressure imaging via background-oriented schlieren tomography: A spatiotemporal measurement for MHz ultrasound fields and hydrophone calibration. Ultrasonics.

[30] Zhu J., Lin G., Hu T., Wang S., Li S., Wang Z., Zhang L. (2025). Ultrasound-assisted multi-physics coupling numerical simulation and hydrophone verification. Ultrasonics.

[31] Gong L., Wright A.R., Hynynen K., Goertz D.E. (2024). Inducing cavitation within hollow cylindrical radially polarized transducers for intravascular applications. Ultrasonics.

[32] Liu J., Huang X., He J. (2026). Acoustic measurement methods and spatiotemporal distribution patterns of microbubble spectra in water under artificial aeration conditions. Ultrasonics.

[33] Ge M., Sun C., Zhang X., Coutier-Delgosha O., Zhang G. (2022). Synchrotron X-ray based particle image velocimetry to measure multiphase streamflow and densitometry. Radiat. Phys. Chem..

[34] Texas Instruments RC4558 Dual General-Purpose Operational Amplifier, Datasheet, Rev. H. https://www.ti.com/lit/ds/symlink/rc4558.pdf.

[35] Boylestad R.L., Nashelsky L. (2013). Electronic Devices and Circuit Theory.

[36] West L.G.M., Simões A.J.R., Teixeira L.d.R., Oliveira L.R., Gomes J.G.d.C., Anjos I.S.M.d., Devesa A.S.B.d.F., Gomes L.R.T.C., Pereira L.G., Neto I.E.L. (2026). High-Speed Microprocessor-Based Optical Instrumentation for the Detection and Analysis of Hydrodynamic Cavitation Downstream of an Additively Manufactured Nozzle. Fluids.

[37] Bendat J.S., Piersol A.G. (2010). Random Data: Analysis and Measurement Procedures.

[38] Press W.H., Teukolsky S.A., Vetterling W.T., Flannery B.P. (2007). Numerical Recipes: The Art of Scientific Computing.

[39] Kinsler L.E., Frey A.R., Coppens A.B., Sanders J.V. (2000). Fundamentals of Acoustics.

[40] Kuttruff H. (2009). Room Acoustics.

[41] Pierce A.D. (2019). Acoustics: An Introduction to Its Physical Principles and Applications.

[42] Blackstock D.T. (2000). Fundamentals of Physical Acoustics.

[43] Wu P., Wang X., Lin W., Bai L. (2022). Acoustic characterization of cavitation intensity: A review. Ultrason. Sonochem..

[44] Ge M., Zhang G., Petkovšek M., Long K., Coutier-Delgosha O. (2022). Intensity and regimes changing of hydrodynamic cavitation considering temperature effects. J. Clean. Prod..

[45] Ge M., Manikkam P., Ghossein J., Subramanian R.K., Coutier-Delgosha O., Zhang G. (2022). Dynamic mode decomposition to classify cavitating flow regimes induced by thermodynamic effects. Energy.

